# Epilepsy and sudden unexpected death in epilepsy in a mouse model of human *SCN1B*-linked developmental and epileptic encephalopathy

**DOI:** 10.1093/braincomms/fcad283

**Published:** 2023-10-20

**Authors:** Chunling Chen, Julie Ziobro, Larissa Robinson-Cooper, Samantha L Hodges, Yan Chen, Nnamdi Edokobi, Luis Lopez-Santiago, Karl Habig, Chloe Moore, Joe Minton, Sabrina Bramson, Caroline Scheuing, Noor Daddo, Katalin Štěrbová, Sarah Weckhuysen, Jack M Parent, Lori L Isom

**Affiliations:** Department of Pharmacology, University of Michigan Medical School, Ann Arbor, MI 48109, USA; Department of Pediatrics, University of Michigan Medical School, Ann Arbor, MI 48109, USA; Department of Pharmacology, University of Michigan Medical School, Ann Arbor, MI 48109, USA; Department of Pharmacology, University of Michigan Medical School, Ann Arbor, MI 48109, USA; Department of Pharmacology, University of Michigan Medical School, Ann Arbor, MI 48109, USA; Department of Pharmacology, University of Michigan Medical School, Ann Arbor, MI 48109, USA; Department of Pharmacology, University of Michigan Medical School, Ann Arbor, MI 48109, USA; Department of Pharmacology, University of Michigan Medical School, Ann Arbor, MI 48109, USA; Department of Pharmacology, University of Michigan Medical School, Ann Arbor, MI 48109, USA; Department of Pharmacology, University of Michigan Medical School, Ann Arbor, MI 48109, USA; Department of Pharmacology, University of Michigan Medical School, Ann Arbor, MI 48109, USA; Department of Pharmacology, University of Michigan Medical School, Ann Arbor, MI 48109, USA; Department of Neurology, University of Michigan Medical School, Ann Arbor, MI 48109, USA; Department of Pediatric Neurology, Charles University and Motol Hospital, V Úvalu 84, 150 06 Prague 5, Czech Republic; Applied & Translational Neurogenomics Group, VIB Center for Molecular Neurology, VIB, Universiteitsplein 1 B-2610 Antwerpen, Belgium; Translational Neurosciences, Faculty of Medicine and Health Science, University of Antwerp, Universiteitsplein 1 B-2610 Antwerpen, Belgium; Department of Neurology, Antwerp University Hospital, Universiteitsplein 1B-2610 Antwerpen, Belgium; µNEURO Research Centre of Excellence, University of Antwerp, Universiteitsplein 1B-2610 Antwerpen, Belgium; Department of Neurology, University of Michigan Medical School, Ann Arbor, MI 48109, USA; Michigan Neuroscience Institute, University of Michigan Medical School, Ann Arbor, MI 48109, USA; Department of Neurology, VA Ann Arbor Healthcare System, Ann Arbor, MI 48105, USA; Department of Pharmacology, University of Michigan Medical School, Ann Arbor, MI 48109, USA; Department of Neurology, University of Michigan Medical School, Ann Arbor, MI 48109, USA

**Keywords:** developmental and epileptic encephalopathy, seizure, sodium channel, β subunit, SUDEP

## Abstract

Voltage-gated sodium channel β1 subunits are essential proteins that regulate excitability. They modulate sodium and potassium currents, function as cell adhesion molecules and regulate gene transcription following regulated intramembrane proteolysis. Biallelic pathogenic variants in *SCN1B*, encoding β1, are linked to developmental and epileptic encephalopathy 52, with clinical features overlapping Dravet syndrome. A recessive variant, *SCN1B-*c.265C>T, predicting *SCN1B*-p.R89C, was homozygous in two children of a non-consanguineous family. One child was diagnosed with Dravet syndrome, while the other had a milder phenotype. We identified an unrelated biallelic *SCN1B-*c.265C>T patient with a clinically more severe phenotype than Dravet syndrome. We used CRISPR/Cas9 to knock-in *SCN1B-*p.R89C to the mouse *Scn1b* locus (*Scn1b^R89/C89^*). We then rederived the line on the C57BL/6J background to allow comparisons between *Scn1b^R89/R89^* and *Scn1b^C89/C89^* littermates with *Scn1b^+/+^* and *Scn1b^−/−^* mice, which are congenic on C57BL/6J, to determine whether the *SCN1B-*c.265C>T variant results in loss-of-function. *Scn1b^C89/C89^* mice have normal body weights and ∼20% premature mortality, compared with severely reduced body weight and 100% mortality in *Scn1b^−/−^* mice. β1-p.R89C polypeptides are expressed in brain at comparable levels to wild type. In heterologous cells, β1-p.R89C localizes to the plasma membrane and undergoes regulated intramembrane proteolysis similar to wild type. Heterologous expression of β1-p.R89C results in sodium channel α subunit subtype specific effects on sodium current. mRNA abundance of *Scn2a*, *Scn3a*, *Scn5a* and *Scn1b* was increased in *Scn1b^C89/C89^* somatosensory cortex, with no changes in *Scn1a*. In contrast, *Scn1b^−/−^* mouse somatosensory cortex is haploinsufficient for *Scn1a*, suggesting an additive mechanism for the severity of the null model via disrupted regulation of another Dravet syndrome gene. *Scn1b^C89/C89^* mice are more susceptible to hyperthermia-induced seizures at post-natal Day 15 compared with *Scn1b^R89/R89^* littermates. EEG recordings detected epileptic discharges in young adult *Scn1b^C89/C89^* mice that coincided with convulsive seizures and myoclonic jerks. We compared seizure frequency and duration in a subset of adult *Scn1b^C89/C89^* mice that had been exposed to hyperthermia at post-natal Day 15 versus a subset that were not hyperthermia exposed. No differences in spontaneous seizures were detected between groups. For both groups, the spontaneous seizure pattern was diurnal, occurring with higher frequency during the dark cycle. This work suggests that the *SCN1B-*c.265C>T variant does not result in complete loss-of-function. *Scn1b^C89/C89^* mice more accurately model *SCN1B*-linked variants with incomplete loss-of-function compared with *Scn1b^−/−^* mice, which model complete loss-of-function, and thus add to our understanding of disease mechanisms as well as our ability to develop new therapeutic strategies.

## Teaser

Chen *et al*. generated a mouse model of the developmental and epileptic encephalopathy variant, *SCN1B-*c.265C>T, predicting *SCN1B*-p.R89C. *Scn1b^C89/C89^* mice more accurately model human variants with incomplete loss-of-function compared with *Scn1b^−/−^* mice, with complete loss-of-function, adding to our translational toolbox to develop novel therapeutic strategies for the genetic epilepsies.

## Graphical Abstract

**Graphical abstract:**
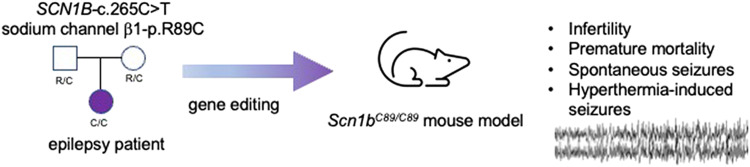

## Introduction

Sodium channelopathies comprise a constellation of central and peripheral nervous system disorders, cardiac arrhythmias and skeletal muscle disorders.^1^ Although the mutated genes and affected cell types are often known, the pathophysiological mechanisms underlying many of these disorders remain poorly understood. One such disorder is Dravet syndrome (DS), a devastating form of developmental and epileptic encephalopathy (DEE) characterized by multiple pharmacoresistant and fever sensitive seizure types, intellectual disability, cognitive decline, movement disorders and increased mortality due to sudden unexpected death in epilepsy (SUDEP).^2,3^ In most cases, DS is caused by *de novo* pathogenic variants in *SCN1A*, encoding the voltage-gated sodium channel (VGSC) Na_v_1.1 α subunit.^4,5^ In addition to *SCN1A*, a growing body of evidence has linked biallelic variants in *SCN1B*, encoding the VGSC β1 and β1B non–pore-forming subunits, to DS or to a more severe disease, early infantile DEE (OMIM DEE52).^6-9^ VGSCs are responsible for generation of the rising phase and propagation of the action potential in mammalian excitable cells.^10^ VGSCs were purified as heterotrimeric complexes of α and β subunits from rat brain.^11^ This work showed that a central α subunit forms the ion-conducting pore and is associated with two different β subunits.^12^ Originally characterized as auxiliary, β subunits are now known to be multifunctional molecules that engage in conducting and non-conducting roles in multiple tissues.^13,14^ During the more than two decades since β subunits were identified, a growing body of research has shown the importance of these proteins not only in normal physiology but also in pathophysiology. The breadth of β1 subunit function hinges on a key structural motif, an extracellular immunoglobulin (Ig) loop, which enables their function as cell adhesion molecules.^13,15,16^ β1 cell adhesion molecule-mediated functions are critical to brain development.^13,15^ Integrity of the Ig loop is also critical for β1-mediated VGSC modulation *in vivo,*^17^ making this domain multi-functional. In their roles as VGSC and voltage-gated potassium channel modulators,^18-22^ β1 subunits make important contributions to the regulation of neuronal firing. As substrates for regulated intramembrane proteolysis (RIP) by β-site amyloid precursor protein cleaving enzyme-1 (BACE1) and γ-secretase, β1 subunits also contribute to the regulation of ion channel gene expression, including genes encoding VGSC α subunits.^22-24^ Considering the diverse roles of VGSC β1 subunits, it is not surprising that variants in *SCN1B* are linked to pathophysiology.

Here, we generated a mouse model of DEE52 using CRISPR-Cas9 (clustered regularly interspaced short palindromic repeats/RNA-guided **Cas9** nuclease) gene editing to introduce the variant, *SCN1B-*c.265C>T, predicting *SCN1B*-p.R89C, located in the β1 extracellular Ig loop domain. This recessive variant was previously found to be homozygous in two children of a non-consanguineous family. One child was diagnosed with DS, while the other had a milder epilepsy phenotype.^25^ Here, we identified an unrelated biallelic *SCN1B-*c.265C>T patient with a clinically more severe phenotype than DS. We asked whether the bilallelic expression of *SCN1B-*c.265C>T *in vivo* results in a phenotype that is similar to *Scn1b*^−/−^ mice, which we have used previously to model DS.^26,27^ Our results show that this novel mouse model partially phenocopies the *Scn1b* null mutation, suggesting that biallelic *SCN1B-*c.265C>T expression does not result in complete *SCN1B* loss-of-function (LOF). Homozygous *SCN1B-*p.R89C mice more accurately model human DEE52 variants with incomplete LOF compared with *Scn1b^−/−^* mice, which model variants with complete LOF, adding to our translational toolbox to develop novel therapeutic strategies for DEE52.

## Materials and methods

### Patient genotyping

Whole exome sequencing was performed for the proband, healthy parents and healthy sibling under IRB approval at the University of Antwerp. Mapping of the reads to the reference genome was done using Burrows–Wheeler Aligner. *De novo* variants were called using DeNovoGear, and the generated list of variants was filtered using the following criteria: read depth in all individuals ≥ 8; allele balance in the proband between 0.25 and 0.75 and in the parents ≥ 0.95; exclusion of variants in tandem repeats and segmental duplications; posterior probability of *de novo* calling of DeNovoGear ≥ 0.5; and exclusion of variants seen in >1 individual. No *de novo* variants were identified in the proband in brain-expressed genes. The dataset was further filtered under a recessive model using the criteria: read depth in all individuals ≥8; allele balance in the proband ≥0.95 and in the parents between 0.25 and 0.75 for filtering under a homozygous model and between 0.25 and 0.75 in the proband and parents for the compound heterozygous model; exclusion of variants in tandem repeats and segmental duplications; and a frequency of ≤1% in control databases.

### *Scn1b* null and littermate mice

All animal procedures in this study were performed in accordance with NIH policy and approved by the University of Michigan Institutional Animal Care and Use Committee. Investigators were blinded to genotype for all experiments. Animals were housed in the Unit for Laboratory Animal Medicine at the University of Michigan Medical School. Male and female pups were used in all experiments, and seizure data were separated by sex, as indicated.

### *Scn1b* null mice

*Scn1b^+/+^* and *Scn1b^−/−^* littermate mice were generated from *Scn1b^+/−^* mice that were congenic on the C57BL/6J background for over 20 N generations.^26^

### Transgenic knock-in mice

CRISPR/Cas9 technology was used to introduce a single amino acid change in exon 3 of Ensembl gene model transcript *Scn1b*-001 (ENSMUSE00000533876). The CRISPOR algorithm^28^ was used to identify two single-guide RNA (sgRNA) targets predicted to cut the chromosome near codon 89: sgRNA C130G1 targeted 5ʹ TGAGCGCTTTGAGGGCCGAG (PAM = TGG) 3ʹ and C130G2 targeted 5ʹ GACTACCGTTCCACACCACT (PAM = CGG) 3ʹ. Phosphorothioate-modified sgRNAs were synthesized by Synthego.^29,30^ Each sgRNA (60 ng/ul) was complexed with enhanced specificity Cas9 protein (ESPCAs9, 30 ng/ul, Millipore-Sigma)^31^ and individually tested to determine if ribonucleoprotein complexes cause chromosome breaks in mouse zygotes. Ribonucleoproteins were microinjected into fertilized mouse eggs. Eggs were placed in culture until they developed into blastocysts. DNA was extracted from individual blastocysts for analysis. PCR with primers spanning the predicted cut site was used to generate amplicons for Sanger sequencing.^32^ Amplicons were produced with C130 forward primer: 5ʹ TTGATCCCATATATGCCTCATCTGTCCTT 3ʹ and C130 reverse primer 5ʹ: CGCTGGTGTTGTGCTCATAATTATCAAAG 3ʹ, resulting in a 329 bp amplicon. Sequencing electropherograms of amplicons from individual blastocysts were evaluated to determine if small insertions/deletions caused by non-homologous endjoining repair of chromosome breaks were present.^33^ sgRNA C130G1 but not C130G2 was found to induce chromosome breaks. C130G1 had a high specificity score of 92.^34^ The use of high specificity sgRNA and high fidelity Cas9 protein has been shown to dramatically reduce the likelihood of off-target hits in mice.^35^

Ribonucleoproteins were mixed with a spot-dialyzed synthetic long single-stranded DNA donor (10 ng/μl, IDT.com) prior to microinjection into mouse zygotes.^36^ The DNA donor was designed to replace wild-type (WT) *Scn1b* codon 89 for arginine (CGA) with a codon for cysteine (TGC) in exon 3. Silent coding changes in the sgRNA binding sequence were included in the oligonucleotide to block cutting by Cas9 after repair of the chromosome by homology-directed repair.^37^ The CRISPR reagents were microinjected into fertilized mouse eggs produced by mating superovulated B6SJLF1 female mice (Jackson Laboratory stock no. 100012) with B6SJLF1 male mice as described.^38^ CRISPR/Cas9 microinjection of zygotes produced potential founder mice. Fifty-one of 112 generation zero (G0) founder pups were identified by Sanger sequencing of amplicons spanning exon 3 into intron 3. Five G0 founders were mated with WT C57BL/6J mice to obtain germline transmission of the *Scn1b*-p.R89C gene. The resulting line was then rederived on a pure C57BL/6J background to be able to compare their phenotype with *Scn1b*^+/+^ and *Scn1b*^−/−^ mice.

The sequence of the single-stranded oligonucleotide DNA donor was as follows:

GAGGGTGACTCATCTGCCCCACTCATCACTCACCACCCTAAGATCCTACGCTATGAGAATGAGGTGCTGCAGCTGGAGGAAGATGAGaGaTTcGAaGGa**tGc**GTGGTGTGGAACGGTAGTCGGGGCACCAAGGACCTGCAGGACCTGTCCATCTTCATCACCAACGTCACCTACAACCACTCTGGCGACTACGAATGTCA

Exon 3 is underlined. The R89C codon is shown in bold. Lower case letters indicate the silent coding changes introduced to block sgRNA binding after repair of a chromosome break with the oligonucleotide donor.

Primers used for genotyping were as follows:

For *Scn1b^R89/R89^* WT:

β1 Endo 5ʹ: 5ʹ-TGA GCG CTT TGA GGG CCG A-3ʹ

β1 Flank 3ʹ: 5ʹ-AGA GAG AAT GGA GAA TCA AGC CAT AG-3ʹ

For *Scn1b^C89/C89^* mutant:

R89C Mut 5ʹ: 5ʹ-GAT GAG AGA TTC GAA GGA TGC-3ʹ

β1 Flank 3ʹ: 5ʹ-AGA GAG AAT GGA GAA TCA AGC CAT AG-3ʹ

Throughout the manuscript, we use the notation *Scn1b^R89/R89^* to indicate WT mice, *Scn1b^R89/C89^* to indicate mice heterozygous for the *SCN1B-*p.R89C mutation and *Scn1b^C89/C89^* to indicate mice homozygous for the *SCN1B-*p.R89C mutation.

### RT-qPCR

Hemispheres of P15 to 18 *Scn1b*^−/−^, *Scn1b^C89/C89^* and *Scn1b^R89/R89^* mouse brains were cut sagitally using a razor blade. The brainstem, cerebellum and hippocampus were then dissected from each hemisphere. To dissect hippocampi, a spatula was used to stabilize the cortex, while a second spatula was placed underneath the ventral part of the hippocampus to separate it from cortical tissue. In a separate group of mice, ∼75 micron coronal slices were made from mouse whole brain, followed by dissection of the somatosensory cortex according to the Allen Brain Atlas. All tissues were dissected in 1× PBS, followed by snap freezing in liquid nitrogen and storage at −8°C. RNA was isolated using the Qiagen RNeasy Plus kit according to the manufacturer’s instructions. Tissue was homogenized with a Tissue-Tearor (BioSpec Products, Inc.) followed by lysis through a sterile, 18-gage hypodermic needle and vortexing. RNA samples were run on a NanoDrop One Spectrophotometer (ThermoFisher Scientific) to ensure adequate concentration and purity and then stored at −80°C. cDNA was generated from 0.75–1.5 μg of RNA using Reverse Transcriptase SuperScript III (RT SS III), random primers (Invitrogen) and dNTPs (Invitrogen). RNA, random primers and dNTPs were incubated at 65°C for 5 min. Salt buffers, 0.1 M DTT, RNase Out and RT SS III were added, and reactions were incubated at 25°C for 5 min, 50°C for 60 min and 70°C for 15 min. cDNA was either diluted 1:3 in RNase-free water or kept undiluted. Quantitative PCR was performed using SYBR Green (Applied Biosystems) and gene-specific primers (Integrated DNA Technologies) on a QuantStudio 7 Flex Real-Time PCR System (Applied Biosystems). Gene-specific measurements of each cDNA sample were run in triplicate, along with the endogenous control gene *Gapdh* or *β-actin* used for normalization and then compared with WT expression levels. The relative expression levels for each gene were quantified using the comparative threshold (2^−ΔΔCt^) method of quantification. Data are presented as the fold change in gene expression ± SEM. Statistical significance (*P* < 0.05) of comparisons between genotypes was determined using a Student’s *t*-test.

### Hyperthermia-induced seizures

Hyperthermia seizure susceptibility was tested at P15 as previously described for *Scn1b^+/−^* mice.^17^ Seizures were classified according to a modified Racine scale.^7,17,39^ After a 1 ml intraperitoneal injection of 0.9% NaCl to prevent dehydration, a rectal thermometer was positioned to monitor body temperature (BT). A heat lamp connected to a temperature monitoring system controlled BT. Mice were acclimated in the chamber at 37.5°C for 30 min. During the observation period, the set temperature (ST) was increased by 0.5°C and then held for 2 min. At the ∼25 min time point, ST was held at 42°C for an additional 15 min. When a seizure was observed, BT, seizure severity (Racine scale) and time elapsed from the beginning of the observation period were recorded. All animals were euthanized at the end of the experiment. Investigators were blinded to genotype. *Scn1b^C89/C89^* and *Scn1b^R89/R89^* mice were compared in each experiment.

In a separate group of animals, we tested whether exposure to hyperthermia at P15, to mimic early-life febrile seizures in DS patients, would sensitize mouse pups to have a higher number of spontaneous seizures as adults. A similar hyperthemia seizure protocol was used as described above but stopped at the point of a Racine scale Grade 5/6 seizure. If Grade 5/6 seizures were not observed, the protocol continued to an ST of 42°C for 5 min and then stopped. Pups were returned to the nest, and video monitored continuously for 2 months followed by implantation of EEG electrodes.

### Video/EEG recording

Screw electrodes were surgically implanted in young adult (P60–90) *Scn1b^R89/R89^* and *Scn1b^C89/C89^* mice. Mice were anaesthetized with isoflurane and placed in a stereotaxic adapter. Bilateral screw electrodes were placed in the skull at approximately anteroposterior = −2.1, mediolateral = +/−1.7, and a common reference electrode was placed over the cerebellum (approximately anteroposterior = −6.0, mediolateral = 0). The electrodes were connected to a 6-pin electrode pedestal, and the headcap was secured using dental cement. After 3–7 days of recovery, simultaneous EEG recordings and infrared video monitoring were performed with a Natus recording system. Signals were acquired at 1024 Hz. Data were filtered with a 1 Hz high-pass filter and 70 Hz low-pass filter. Seizures and interictal background were assessed manually by an experienced reader. Seizures were defined as a sudden burst of electrographic activity consisting of rhythmic spike-and-wave discharges lasting >10 s and evolving in frequency and amplitude. Interictal epileptiform discharges were defined as transients distinguishable from background activity with a characteristic morphology as defined by Kane *et al.*^40^ The presence of IED was noted, but they were not quantified.

### Western blot analysis

Brain membrane proteins were prepared from P60 to 90 mice as described.^41^ Complete protease inhibitors (Roche Diagnostics) were added to all solutions at twice the recommended concentration to minimize protein degradation. Deglycosylation of membrane protein samples, where indicated, was performed using PNGaseF (New England BioLabs Cat. #P0704S) as previously described.^17^ For western blotting, 50–80 µg aliquots of membrane protein were separated by sodium dodecyl-sulfate polyacrylamide gel electrophoresis (SDS-PAGE) and processed for western blotting with anti-β1_intra_ antibody (1:1000) (Cell Signaling Technologies 13950) or anti-Na_V_1.1 Na^+^ channel antibody (K74/71) (1:200) (NeuroMab 75-023) as previously described.^22^ Anti-α-tubulin (1:1000) (Cedar Lane) or anti-transferrin receptor (TfR) (1:200) (Invitrogen H68.4 13-6800) antibodies were used as loading control, as indicated. Immunoreactive bands were detected using Supersignal West Dura Extended Duration Substrate (Therma Scientific #34076) and imaged using an iBrightFL1000 system (Invitrogen). Immunoreactive signals for the deglycosylated bands were quantified using iBright analysis software (Invitrogen) and normalized to the level of α-tubulin for each sample.

### Cell surface biotinylation

Previously generated stable Chinese hamster lung (CHL) cell lines expressing WT β1V5 or β1-p.R89C-V5^22^ were grown in 150-mm tissue culture plates until 90–100% confluent. Cell surface proteins were biotinylated using the Cell Surface Biotinylation and Isolation Kit (Pierce, Cat#A44390) according to the manufacturer’s protocol, except that the biotinylation reaction was performed at 4°C for 30 min without mechanical agitation. Loading buffer containing 1% sodium dodecyl sulphate, 1 mmol/L β-mercaptoethanol and 0.2% dithiothreitol were added to samples and heated for 8 min at 85°C. Samples were separated on 10% tris-glycine polyacrylamide gels, transferred to polyvinylidene difluoride membrane (16 h, 55 mA, 4°C) and probed with antibodies. Membranes were probed with three primary monoclonal mouse antibodies: anti-V5 (1:1000 dilution, Invitrogen 46-0705), anti-HSP90 (1:500 dilution, Enzo Scientific AC88) and anti-TfR (1:500, Invitrogen H68.4). Mouse HRP-conjugated secondary antibodies were utilized (1:1000 dilution for anti-TfR, anti-V5 and anti-HSP90). Primary antibody incubations were performed overnight at 4°C, followed by secondary antibody incubation at RT for 1 h. Antibodies were diluted in 5% milk and 1% BSA in TBST. Secondary antibody was diluted in 5% milk and 1% BSA in TBST and then incubated for 1 h at RT. Immunoreactive bands were detected using SuperSignal West Dura Extended Duration Chemiluminescent Substrate (Thermo Scientific Ref#34076) and imaged on an iBright FL1000 (Invitrogen) within the linear range of the instrument by utilizing the iBright Smart Exposure feature.

### Cleavage assay

Stable CHL cell lines expressing WT β1V5 or β1-p.R89C-V5 were grown until ∼70% confluent in 100 mM tissue culture plates. Cells were treated with vehicle (0.1% DMSO), or the γ-secretase inhibitor Avagacestat (10 μm), or the γ-secretase inhibitor L-685,458 (10 μm) for 24 h. Twenty-four hours post-treatment, cells were harvested and whole cell lysates were prepared. Briefly, harvested cell pellets were resuspended in 50 mM Tris, pH 8.0 with Complete protease inhibitors, EDTA-Free (Roche) and 1 mM Na_3_VO_4_. Cells were incubated on ice for 30 min and sonicated 3× at 20% power for 10 pulses every 10 min. Lysates were centrifuged at 14 000×g for 5 min to remove large insoluble fragments, followed by the supernatant being removed and stored at −8°C. Samples were separated on 12% SDS-PAGE gels, and western blots were performed as described above.

### Whole-cell patch clamp analysis of transfected human embryonic kidney cells

Stable human embryonic kidney (HEK) cell lines expressing human Na_v_1.1 (a gift from Dr M. Mantegazza), Na_v_1.5 (a gift from Dr J. Makielski) or Na_v_1.6 (a gift from Essen Bioscience) were maintained at 37°C and 5% CO2 in Dulbecco’s Modified Eagle Medium supplemented with 5% heat-inactivated fetal bovine serum (Corning), 100 U/ml penicillin/streptomycin (Gibco) and 600 µg/ml G418 (Gibco). For electrophysiological analyses, HEK-Na_v_1.1, -Na_v_1.5 or -Na_v_1.6 cells were transiently transfected with β1-WT-V5-2AeGFP (green fluorescent protein), β1-p.R89C-V5-2AeGFP or enhanced GFP (eGFP) (1 µg of cDNA with 5 µl of Lipofectamine 2000). Inclusion of the 2AeGFP sequence provided a cleaved, eGFP fluorescent marker to identify transfected cells during electrophysiological recording. After 12–24 h, cells were split to a lower density in 35-mm dishes, and GFP-positive HEK cells were identified by epifluorescence for whole-cell voltage-clamp recording by an investigator blind to genotype. Each electrophysiology experimental figure represents data from three or more separate transfections.

Electrophysiological recordings were performed ∼12 h following final plating. Sodium current (*I_Na_*) was recorded from GFP-positive HEK cells in the presence of an external solution containing (in mM): 120 NaCl, 1 BaCl_2_, 2 MgCl_2_, 0.2 CdCl_2_, 1 CaCl_2_, 10 HEPES, 20 TEA-Cl and 10 glucose (pH = 7.35 with CsOH, osmolarity = 300–305 mosm). Fire polished pipettes with resistance of 1.5–3 mΩ were filled with an internal solution containing (in mM): 1 NaCl, 150 N-methyl-d-glucamine, 10 ethylene glycol tetraacetic acid (EGTA), 2 MgCl_2_, 40 HEPES, 25 phosphocreatine-tris, 2 MgATP, 0.02 Na_2_GTP and 0.1 leupeptin (pH = 7.2 with H_2_SO_4_). All recordings were performed within 10–60 min after the culture medium was replaced by the external recording solution, and the dish with cells was placed on the recording setup. Holding potential was −80 mV.

### Statistical analysis

Kaplan–Meier (Wilcoxon) plots were used to analyse mouse survival, temperature at first seizure and latency to first seizure. Quantitative reverse transcription polymerase chain reaction (Rt-qPCR) results between genotypes were analysed using Student’s *t*-test. Anti-Nav1.1 western blotting results between genotypes were analysed using Student’s *t*-test, with relative Na_V_1.1 intensity being normalized to vehicle expression [anti-TfR] and compared with *Scn1b^+/+^* mice. Data are represented as the mean ± SEM. Seizure frequency during the light and dark cycles was analysed using the paired sample one-tailed *t*-test. All analyses, with the exception of electrophysiology, were performed using GraphPad Prism 9.0 software. Voltage clamp analysis was performed using pClamp 11 (Molecular Devices) and SigmaPlot 11 (Systat software). Statistical analyses, *t*-test, and one-way ANOVA, were performed using SigmaPlot. For all experiments, significance is defined as *P*-value <0.05.

## Results

### Patient phenotype

The proband was born after a normal pregnancy from healthy unrelated parents at the 40th week of gestation. Birth weight was 3650 g and birth length 50 cm. Post-natal adaptation was normal, but developmental milestones were delayed. Rolling occurred at 5–6 months, with independent walking at 18 months, which worsened over time. At the age of 11 years, the patient was able to take a few independent steps indoors but did not walk outdoors. The patient’s gait is ataxic and crouched. The first words of speech occurred around 12 months of age. By 11 years of age, the patient was able to repeat words but did not speak spontaneously. The patient requires dressing and feeding and is not toilet trained. Mental deterioration is evident, and behavioural problems with autistic features and aggression are present. Neurological exam showed microcephaly, ataxia, central hypotonia and severe intellectual disability. No focal neurological signs were present. Metabolic screening was normal. Brain MRI showed a small arachnoid cyst and diffuse white matter changes and delayed myelination at the age of 14 months. Mild signs of delayed myelination next to the lateral ventricles were observed at the age of 3.5 years.

*Epilepsy:* The first clonic seizures of upper limbs appeared at the age of 6 months at the beginning of febrile infections. At the age of 7 months, seizures with clonic jerking evolved into status epilepticus. Chronic medication with phenobarbital was started. In the following months, the patient had frequent seizures with mostly symmetric clonic jerking of limbs or seizures with loss of consciousness without convulsions. Prolonged seizures required sedation at the intensive care unit. Seizures always occurred at the beginning of illness with increased BT detected after the seizure. Afebrile seizures were not observed until 2 years of age. In the third year of life, the patient had tonic-clonic seizures and many myoclonic jerks while treated with lamotrigine, topiramate and valproic acid. Seizure semiology changed over the years. In addition to clonic and tonic-clonic seizures, the patient had hypomotor seizures, atypical absences with staring and perioral cyanosis, hypotonic drop attacks and seizures with vegetative symptoms including mydriasis, loud outcry and perioral and acral (hands and earlobe) cyanosis. Prolonged tonic-clonic seizures and clusters of seizures occurred during febrile illnesses, and intensive care unit admission was needed repeatedly. EEG was normal both in wakefulness and sleep in the first year, moderate slowing of the background appeared in the second year and sporadic bifrontal spikes were seen in the third year of age with high-voltage spike wave complexes in the left fronto-centro-parietal area. Background EEG remained abnormal with slow activity and sporadic spiking in the frontal areas. Generalized spikes were captured only in sleep. Antiseizure medications failed to control seizures over the long term despite trying various combinations of phenobarbital, valproic acid, topiramate, clonazepam, stiripentol and primidone. Vagal nerve stimulation was implemented at the age of 7 years resulting in a temporary decrease in seizure frequency.

Genetic diagnosis identified a homozygous variant in *SCN1B*, c.265C>T, predicting p.R89C (NM_199037) in the proband, with parents and sibling as heterozygous carriers. The variant was seen twice in the Exac database (allele frequency 1.647 × 10^−05^) and was validated with Sanger sequencing. A diagnosis of DS was made at the age of 3.5 years.

### Generation and characterization of transgenic mice

**Figure 1:**
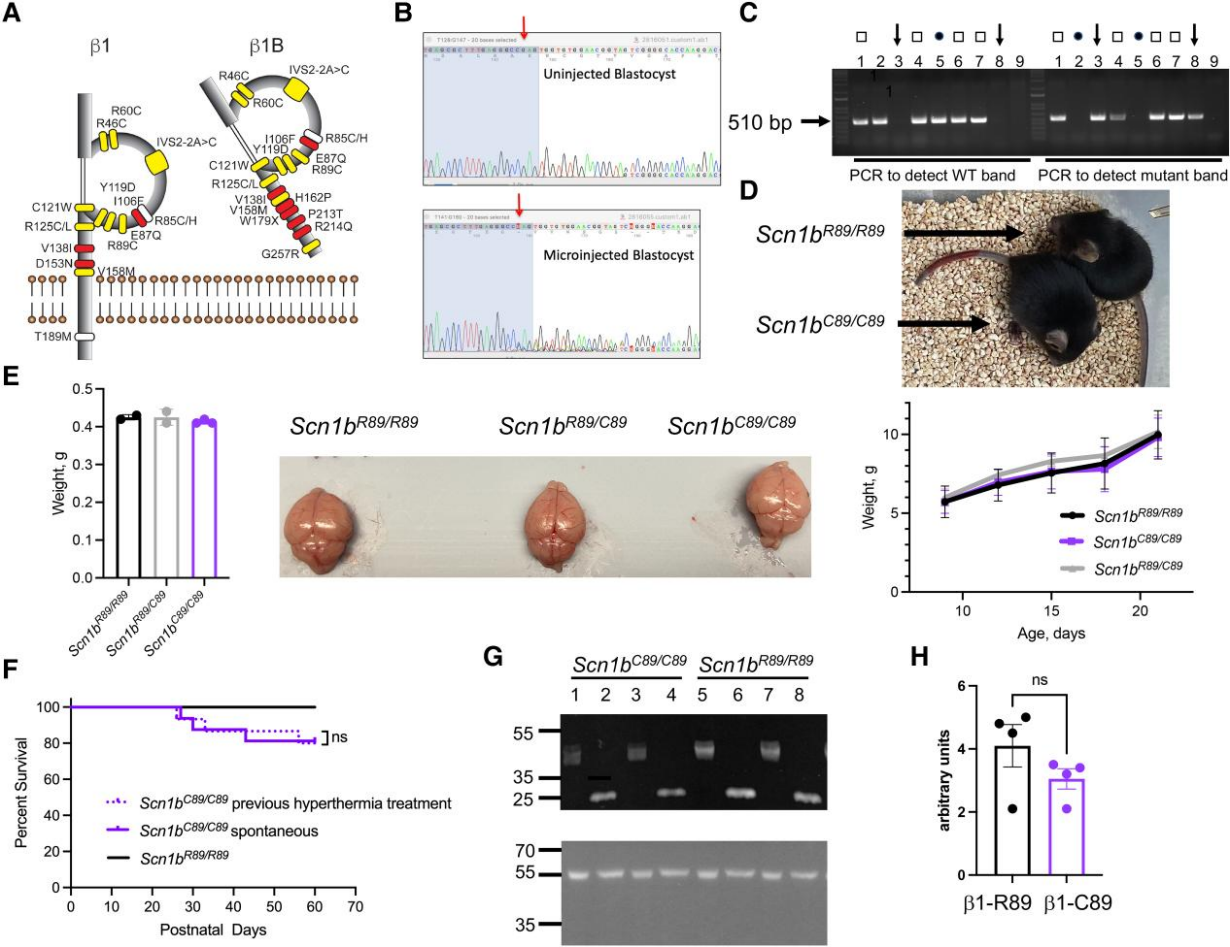
**Generation and characterization of *Scn1b^R89/C89^* mice. (A)** Known disease variants in VGSC β1 and β1B subunits. Adapted from O’Malley *et al.*^15^ Yellow: variants associated with epilepsy. White: variants associated with epilepsy and cardiac arrhythmia. Red: variants associated with sudden cardiac death. Residue R89, yellow, is located within the Ig loop domain of β1 and β1B and is evolutionarily conserved throughout vertebrate VGSCs (GeneCards The Human Gene Database, https://media.githubusercontent.com/media/aminodektc/70/master/SCN1B/SCN1B.png). (**B)** sgRNAs were complexed with ESPCas9 protein and injected into fertilized mouse eggs. A DNA genomic fragment spanning the expected Cas9 cut site was PCR amplified and sequenced analysis. Cas9-induced double-strand breaks resulted in the presence of superimposed sequences (peaks-on-peaks) starting near the expected Cas9 cut site. sgRNA C130G1 produced indel mutations in three of five test blastocysts. Arrow: Cas9 cut site. Blue-shaded nucleotides: sgRNA target. (**C)** Representative genotyping experiment in which two separate PCRs were run for each tail DNA for *Scn1b^R89/R89^*, *Scn1b^R89/C89^* and *Scn1b^C89/C89^* pups to detect WT and mutant *Scn1b* bands, respectively. Lanes 1, 4, 6 and 7: *Scn1b^R89/C89^* and show both WT and mutant bands (squares); Lanes 2 and 5: *Scn1b^R89/R89^* and show WT bands only (circles); Lanes 3 and 8: *Scn1b^C89/C89^* and show mutant bands only (arrows). (**D)** Upper: Comparison of littermate P19 *Scn1b^R89/R89^* and *Scn1b^C89/C89^* animals. Lower: Comparison of *Scn1b^R89/R89^* (*N* = 3), *Scn1b^R89/C89^* (*n* = 5) and *Scn1b^C89/C89^* (*n* = 5) male and female weights from P9 to P21. No significant differences between genotypes (mean +/− SD, one-way ANOVA). **(E)** Left: Comparison of whole brain weights per genotype at P21 showing no statistical differences (mean +/− SD, one-way ANOVA). Right: Comparison of acutely dissected brains of littermate *Scn1b^R89/R89^*, *Scn1b^R89/C89^* and *Scn1b^C89/C89^* animals at P21. (**F)** Kaplan–Meier analysis of life span shows that ∼20% of *Scn1b^C89/C89^* animals die by P60. Dotted purple line: *Scn1b^C89/C89^* animals pre-treated with hyperthermia at P15 and then allowed to develop (*n* = 15). Solid purple line: non–pre-treated *Scn1b^C89/C89^* animals (*n* = 16). Solid black line: non–pre-treated *Scn1b^R89/R89^* animals (*n* = 31). No significant differences between pre-treated and non–pre-treated groups (ns, Log-rank Mantel–Cox test). Male and female animals were included in each group. Original, uncropped blots shown in Supplementary Fig. 1. (**G)** Expression of β1 polypeptides in P60-90 *Scn1b^R89/R89^* and *Scn1b^C89/C89^* brains. Lanes 1–4: *Scn1b^C89/C89^*. Lanes 5–8: *Scn1b^R89/R89^*. Odd numbered samples are untreated and thus glycosylated. Even numbered samples were deglycosylated with PNGaseF. (**H)** Densitometric quantification of deglycosylated β1 polypeptides from *Scn1b^R89/R89^* (β1-R89) and *Scn1b^C89/C89^* (β1-C89) brains. Deglycosylated β1 (∼22 kDa) immunoreactive bands were normalized to loading control anti–α-tubulin signal. *n* = 4 *Scn1b^R89/R89^* and 4 *Scn1b^C89/C89^* samples. No significant differences between groups (mean +/− SEM, *P = 0.2*, unpaired *t*-test).

The residue R89 is located within the Ig loop domain of VGSC β1 and β1B subunits (Fig. 1A) and is evolutionarily conserved throughout vertebrate *Scn1b* genes (GeneCards: The Human Gene Database, https://media.githubusercontent.com/media/aminodektc/70/master/SCN1B/SCN1B.png). To evaluate the *SCN1B-*c.265C>T variant *in vivo*, knock-in mice expressing the mutation were generated using CRISPR/Cas9 technology. Cas9-induced double-strand breaks resulted in the presence of superimposed sequences (peaks-on-peaks) starting near the expected Cas9 cut site (Fig. 1). sgRNA C130G1 produced indel mutations in three of five test blastocysts (Fig. 1B). CRISPR/Cas9 microinjection of zygotes produced potential founder mice. Twenty-nine of 112 G0 founder pups were identified by Sanger sequencing of amplicons spanning exon 3 and loxP sites. Five G0 founders were mated with WT C57BL/6J mice to obtain germline transmission of the *Scn1b*-p.R89C alelle. One of the resulting lines (line 4) was then rederived on a pure C57BL/6J background to be able to compare their phenotype with *Scn1b*^+/+^ and *Scn1b*^−/−^ mice, which are congenic for over 20 N generations on C57BL/6J. *Scn1b^R89/C89^* mice had normal lifespans, body weights and fertility with no observed behavioural seizures. In contrast, *Scn1b^C89/C89^* mice were infertile. Thus, all experimental mice were generated from the mating of heterozygous *Scn1b^R89/C89^* littermates. The yield of a subset of *Scn1b^R89/R89^*, *Scn1b^R89/C89^* and *Scn1b^C89/C89^* offspring from these matings was 29:48:22, an approximate Mendelian ratio of 1:2:1. Figure 1C shows a representative genotyping experiment in which two separate PCRs were run for each tail DNA sample for *Scn1b^R89/R89^*, *Scn1b^R89/C89^* and *Scn1b^C89/C89^* offspring to detect WT and/or mutant *Scn1b* bands, respectively, as indicated. Figure 1D (top) shows littermate *Scn1b^R89/R89^* and *Scn1b^C89/C89^* animals at P19. Figure 1D (bottom) compares animal weights from P9 to P21, showing no significant differences between genotypes. Because the proband showed microcephaly, we compared brain weights between genotypes at P21. Figure 1E (left) shows no significant differences between genotypes for brain weights. Examples of brains from each genotype taken from a single litter at P21 are show in Fig. 1E (right). Kaplan–Meier analysis of mouse life span shows that ∼20% of *Scn1b^C89/C89^* animals undergo premature death by ∼P60 (Fig. 1F, solid purple line), while *Scn1b^R89/R89^* animals have normal life spans (Fig. 1F, solid black line). Because the proband had frequent febrile seizures as an infant, we induced a Racine scale Grade 5/6 seizure in P15 *Scn1b^C89/C89^* pups using a hyperthermia protocol and then placed them back in the nest to develop with their littermates. Kaplan–Meier analysis of life span for hyperthermia pre-treated animals was not different from untreated *Scn1b^C89/C89^* animals (Fig. 1F, dotted purple line).

*Scn1b^C89/C89^* mouse brains have similar overall levels of β1 protein expression compared with *Scn1b^R89/R89^* littermates. Figure 1G, upper panel, compares β1 polypeptide abundance in brain membranes prepared from 4 *Scn1b^R89/R89^* and 4 *Scn1b^C89/C89^* P60–90 mice compared with an anti-α-tubulin loading control (lower panel). Anti-β1 antibody detected multiple immunoreactive bands for *Scn1b^R89/R89^* and *Scn1b^C89/C89^* mice, in agreement with previous data showing differential glycosylation of β1 polypeptides *in vivo.*^17,42,43^ Deglycosylation of β1 polypeptides using PNGaseF collapsed these bands to a single band of ∼22 kDa for both genotypes (Fig. 1G, upper panel). Because quantification of the multiple glycosylated β1 species is unreliable, we used densitometry to quantify the deglycosylated anti-β1 immunoreactive bands relative to the α-tubulin loading control for each sample (Fig. 1H). While these data showed a trend towards lower expression of β1-p.C89 protein in mouse brain compared with WT, there were no significant differences between average values.

### β1 and β1-p.R89C polypeptides localize to the plasma membrane and are substrates for regulated intramembrane proteolysis in heterologous cells

**Figure 2:**
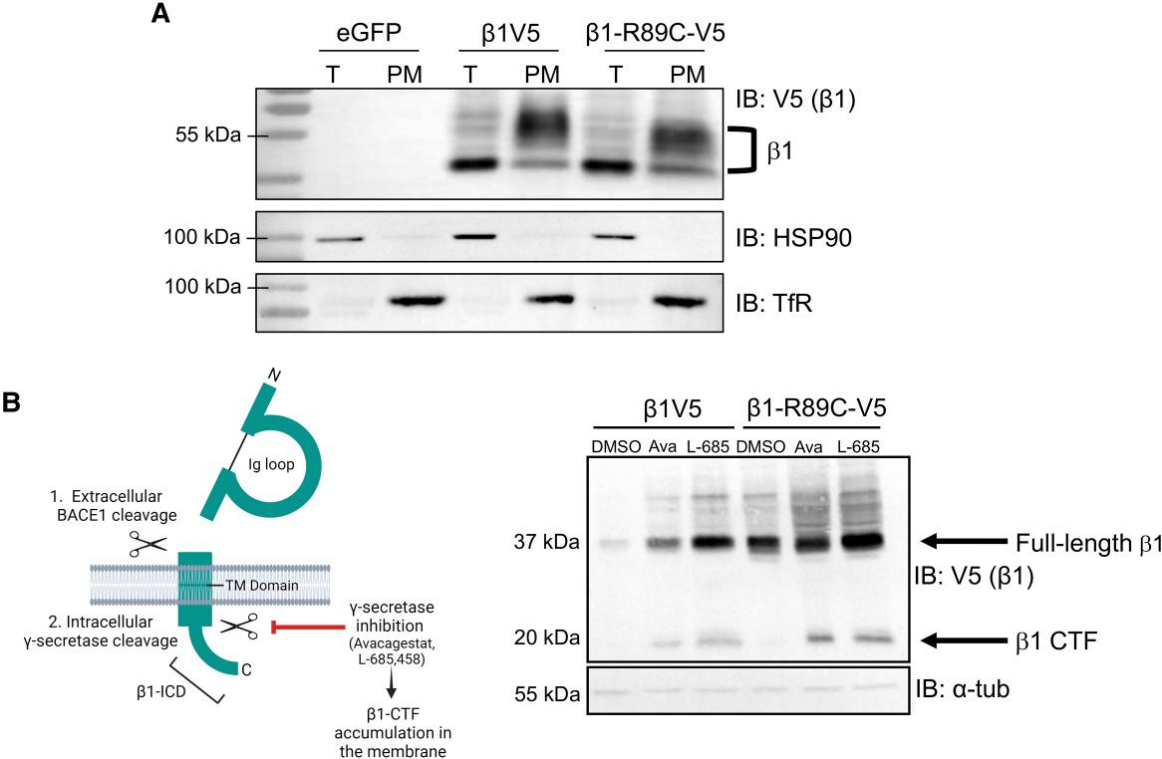
**β1 and β1-p.R89C polypeptides localize to the plasma membrane and are substrates for RIP in heterologous cells. (A)** Cell surface biotinylation shows that β1-p.R89C localizes to the plasma membrane similarly to WT β1, indicated by the presence of β1-p.R89C-V5 and β1V5 in the total cell lysate (T) and PM fraction. Total protein and neutravidin-selected cell surface proteins were analysed by western blot with anti-V5 antibody. Anti-HSP90 antibody was used as a control to ensure biotinylation of cell surface but not intracellular proteins. Anti-transferrin receptor (TfR) antibody was used as a control to ensure that only cell surface proteins were pulled down in the neutravidin selection. *n* = 3. β1 immunoreactive bands are indicated at ∼37 kDa and above, representing various levels of avidin attachment, as shown in our previous work.^7^ Original, uncropped blots shown in Supplementary Fig. 2. **(B)** Left: Cartoon summarizing β1 RIP by BACE1 and γ-secretase as well as γ-secretase inhibition by small molecules. Right: CHL cells stably expressing β1V5 or β1-p.R89C-V5 were used in cleavage assays to determine whether β1-p.R89C undergoes RIP similar to WT β1. Treatment with γ-secretase inhibitor, Avagacestat (10 μM) or L-685,458 (10 μM), for 24 h resulted in accumulation of the β1-CTF (∼20 kDa, arrow) in both cell lines, indicating cleavage of β1. Results are representative of three independent experiments. Original, uncropped blots shown in Supplementary Fig. 3.

Localization to the plasma membrane is required for VGSC β1 subunit-mediated channel regulation and cell adhesion. To determine whether β1-p.R89C localizes to the cell surface, we performed cell surface biotinylation experiments using CHL cell lines that stably overexpress the polypeptides β1V5 or β1-p.R89C-V5. These stable cell lines were previously established with cDNA expression vectors containing a carboxyl-terminal in-frame V5 epitope tag, a cleaving 2A sequence and eGFP.^22^ Anti-HSP90 antibody was used as an intracellular control to ensure that only cell surface proteins were biotinylated, and anti-TfR antibody was used as confirmation that cell surface proteins were enriched in the plasma membrane fraction. We found that β1-p.R89C polypeptides localize to the cell surface similar to β1V5, demonstrated by the presence of anti-V5 immunoreactive bands corresponding to β1-p.R89C-V5 or β1V5, respectively, in the total cell lysate (T) and plasma membrane (PM) fraction (Fig. 2A). β1 immunoreactive bands are indicated in the figure at ∼37 kDa and above, representing various levels of avidin attachment, as shown in our previous work.^7^

VGSC β1 subunits are substrates for RIP.^23^ We previously demonstrated that β1 undergoes sequential cleavage by BACE1 and γ-secretase, resulting in the generation of a soluble intracellular domain (β1-ICD) that can translocate to the nucleus and regulate transcription^22,23^ (Fig. 2B, left panel). To determine whether β1-p.R89C is also a substrate for RIP, we performed cleavage assays in β1V5 or β1-p.R89C-V5 stable CHL cells. CHL cells are optimal for VGSC β1 heterologous RIP studies because they do not express endogenous *Scn1b* mRNA but do express endogenous low levels of BACE1 and γ-secretase.^22^ Treatment of cells with the γ-secretase inhibitor Avagacestat (10 μM) or L-685,458 (10 μM) for 24 h resulted in accumulation of the β1-carboxyl-terminal fragment (β1-CTF), visible at ∼20 kDa on the western blot, compared with vehicle treatment (DMSO), for both cell lines (Fig. 2B, right panel). These results show that both WT β1 and β1-p.R89C undergo RIP *in vitro*.

### β1-p.R89C shows differential regulation of voltage-gated sodium channel α subunit generated *I_Na_* in heterologous cells

**Figure 3:**
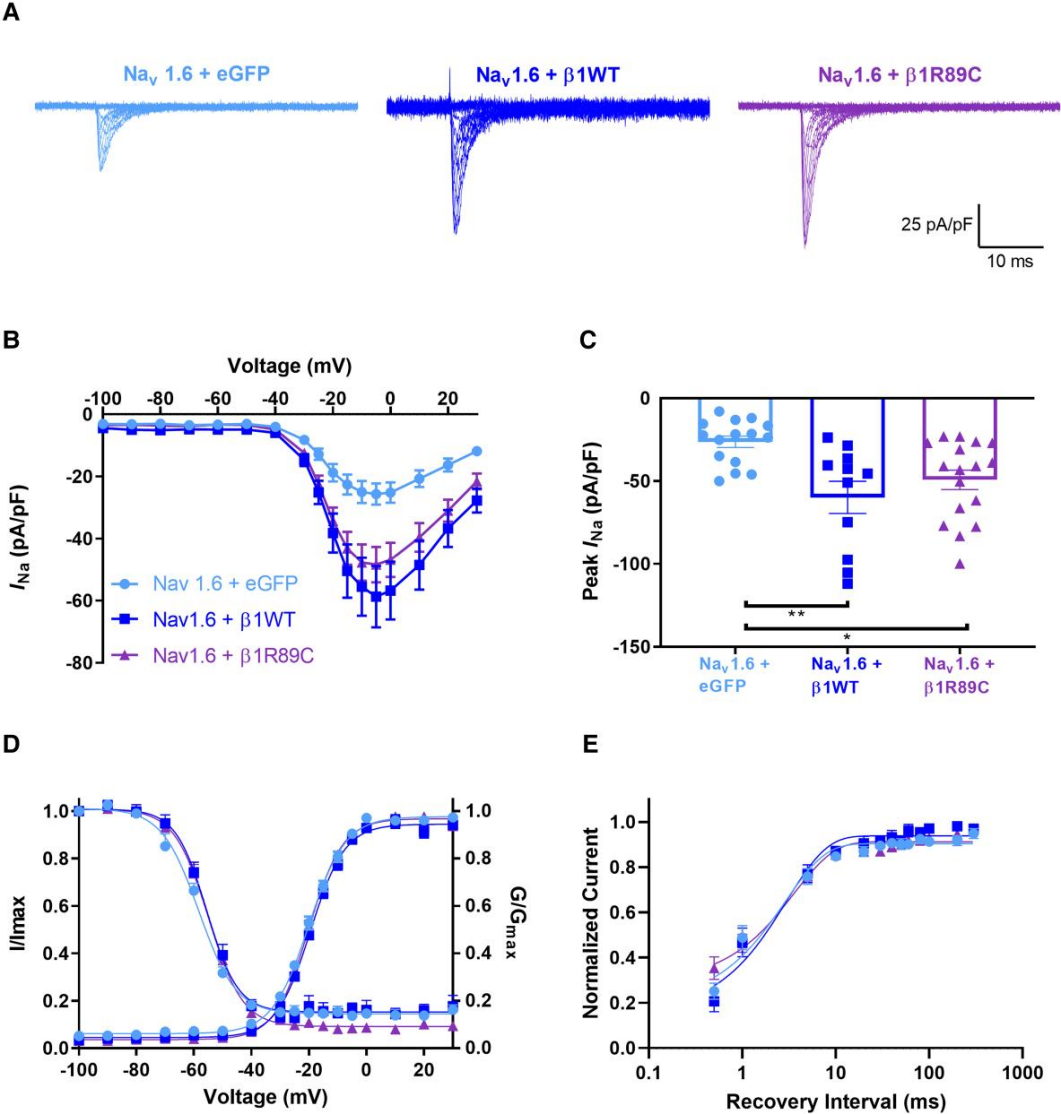
**β1-p.R89C modulates Na_v_1.6-generated *I*_Na_ density.** HEK cells stably expressing human Na_v_1.6 were transiently co-transfected with β1-WT-V5-2AeGFP (dark blue) β1-p.R89C-V5-2AeGFP (purple), or eGFP (light blue). Cells transfected with eGFP were used as negative controls. (**A**) Representative *I*_Na_ density traces. (**B**) Na_v_1.6 *I*_Na_ current–voltage relationship. (**C**) *I_Na_* density was increased with co-expression of WTβ1 or β1-p.R89C. (**D**) No differences in the mean voltage-dependent activation and inactivation curves were observed. (**E**) Recovery from inactivation was expressed as the fraction of current produced by a second pulse over time following an identical pre-pulse. The data were fit to a double exponential function. Data in (**B**), (**C**), (**D**), and (**E**) are presented as means ± SEM. **P < 0.01, *P < 0.05 by a one-way ANOVA with Tukey’s *post hoc* comparison test. Dots represent individual cells. Voltage-dependence of activation and voltage-dependence of inactivation, *n* = 17 (eGFP alone), 16 (+β1), 14 (+β1-p.R89C); recovery from inactivation, *n* = *n* = 8 (eGFP alone), 6 (+β1), 6 (+β1-p.R89C).

*I*_Na_ density is normally increased by heterologous co-expression of β1 subunits via their chaperone function of VGSC α subunits to the plasma membrane (reviewed in^44^). *SCN1B*-linked channelopathy variants have shown abnormalities in modulation of *I_Na_* density and voltage-dependent properties when expressed in heterologous cells.^7,45-49^ Here, we assessed the effects of β1-p.R89C co-expression on *I_Na_* density expressed by the tetrodotoxin-sensitive channels, Na_v_1.1 or Na_v_1.6, or the tetrodotoxin-resistant channel, Na_v_1.5. We co-transfected eGFP (control, light blue), β1-WT-V5-2AeGFP (dark blue) or β1-p.R89C-V5-2AeGFP (purple) into HEK cells stably expressing human Na_v_1.1 (Supplementary Fig. 4), human Na_v_1.6 (Fig. 3) or human Na_v_1.5 (Supplementary Fig. 5) cDNAs. As expected, β1-WT co-expression with all three channels resulted in significantly increased transient *I*_Na_ density (*P* < 0.001) compared with α alone (eGFP) (Supplementary Figs. 4B, 3B and 5B). β1-p.R89C co-expression with Na_v_1.1 or Na_v_1.5 had no effect on transient *I*_Na_ density (Supplementary Figs. 4B and 5B). In contrast, β1-p.R89C co-expression with Na_v_1.6 resulted in increased transient *I*_Na_ density (*P* < 0.001) compared with α alone (eGFP) (Fig. 3B). Neither β1-WT nor β1-p.R89C significantly modulated the voltage dependence or kinetic properties of Na_v_1.1-, Na_v_1.5- or Na_v_1.6-generated *I_Na_* (Supplementary Figs. 4D, 4E, 5D and 5E; Supplementary Tables 1–3; Fig. 3D and E). These results show that β1-p.R89C polypeptides differentially modulate VGSC α subunits *in vitro*.

### *Scn1b^−/−^* and *Scn1b^C89/C89^* mouse brains show differential abundance of voltage-gated sodium channel α subunit mRNAs

Following RIP, the β1-ICD can translocate to the nucleus and regulate expression of several subsets of genes, including those encoding voltage-gated sodium, potassium and calcium channel subunits.^22^ We asked whether β1 and β1-p.R89C regulate similar VGSC α subunit genes by comparing the abundance of *Scn1a*, *Scn2a*, *Scn3a*, *Scn4a*, *Scn5a*, *Scn8a* and *Scn9a* mRNAs in *Scn1b^−/−^* and *Scn1b^C89/C89^* mouse brain areas using RT-qPCR.

**Figure 4:**
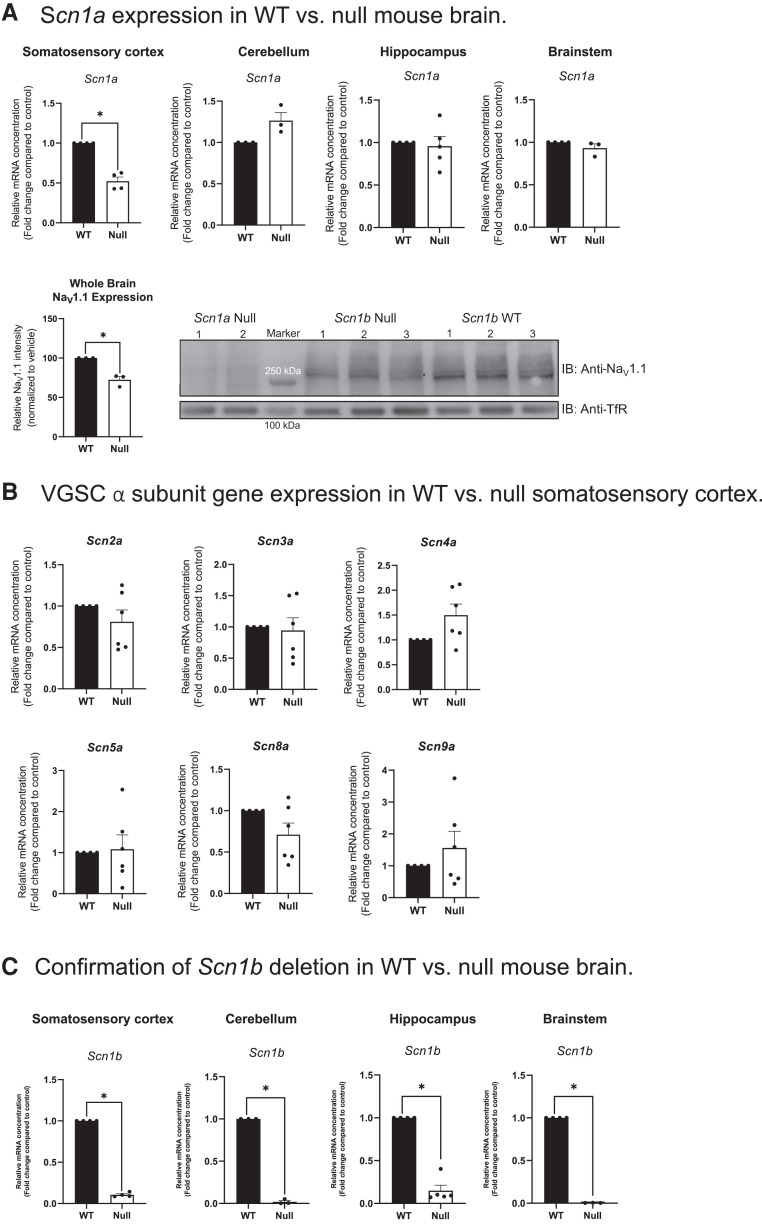
**Differential VGSC α and β subunit expression in P15-18 *Scn1b^+/+^* and *Scn1b^−/−^* mouse brains. (A)** S*cn1a* and Nav1.1 expression in *Scn1b^+/+^* (WT) versus *Scn1b^−/−^* (null) mouse brain. *Scn1a* gene expression was significantly decreased in null somatosensory cortex (*P < 0.001); however, no changes in *Scn1a* were detected in the cerebellum, hippocampus or brainstem (*P > 0.05*). Bottom panel: Nav1.1 protein expression was significantly decreased in *Scn1b* null mouse whole brain membranes compared with WT whole brain. Left: Quantification of anti-Nav1.1 immunoreactive bands normalized to corresponding anti-TfR bands for *Scn1b* WT versus null brains for the blot shown on the right. Data are represented as means ± SEM for three WT and three null brains, respectively. Statistical significance was determined using Student’s *t*-test (*P < 0.01). Right: Western blot analysis of Nav1.1 protein in *Scn1b* null and WT whole brain membranes, as indicated. Upper blot: anti-Nav1.1. Lower blot: anti-TfR. Molecular weight markers are indicated. **(B)** VGSC α subunit gene expression in WT versus null somatosensory cortex. No changes were detected in the relative expression of *Scn2a*, *Scn3a*, *Scn4a*, *Scn5a*, *Scn8a* or *Scn9a* between null and WT somatosensory cortex (*P > 0.05*). **(C)** Confirmation of *Scn1b* deletion in WT versus null mouse brain. Relative expression of *Scn1b* in null and WT mouse somatosensory cortex, cerebellum, hippocampus and brainstem (*P* < 0.0001). Statistical significance was determined using Student’s *t*-test (*P*-value < 0.05). Data are represented as the mean ± SEM. WT: n = 3–5, null: n = 3–5. Male and female mice were used in all experiments (A and B).

We used *Scn1b^−/−^* mice previously to investigate genes that are normally regulated by the β1-ICD in heart.^22^ Here, we found that *Scn1a*, encoding Na_V_1.1, mRNA abundance was reduced by ∼50% in the somatosensory cortex of *Scn1b^−/−^* brain compared with *Scn1b^+/+^* (*P* < 0.001), with no changes detected in the cerebellum, hippocampus or brainstem (*P* > 0.05) (Fig. 4A). This change in the somatosensory cortex was specific to *Scn1a*, as neither *Scn2a*, *Scn3a*, *Scn4a*, *Scn5a*, *Scn8a* nor *Scn9a* α subunit mRNAs showed altered abundance in this brain area (Fig. 4B). In addition, Na_V_1.1 protein expression was reduced ∼30% in *Scn1b*^−/−^ whole brain compared with *Scn1b*^+/+^ whole brain, as assessed by western blot (*P* < 0.01) (Fig. 4A). Confirmation of the near absence of *Scn1b* mRNA in the *Scn1b^−/−^* mouse model is shown in Fig. 4C (*P* < 0.0001). Taken together, these results suggest that the ICD generated from WT β1 normally regulates *Scn1a* mRNA abundance in mouse somatosensory cortex. Furthermore, the observation of *Scn1a* haploinsufficiency resulting from *Scn1b* deletion suggests an additive mechanism for the severity of the *Scn1b^−/−^* model compared with *Scn1a^+/−^* DS mice, which have a later age of seizure onset and a lower rate of SUDEP^50^ compared to *Scn1b^−/−^* mice.^26^

**Figure 5:**
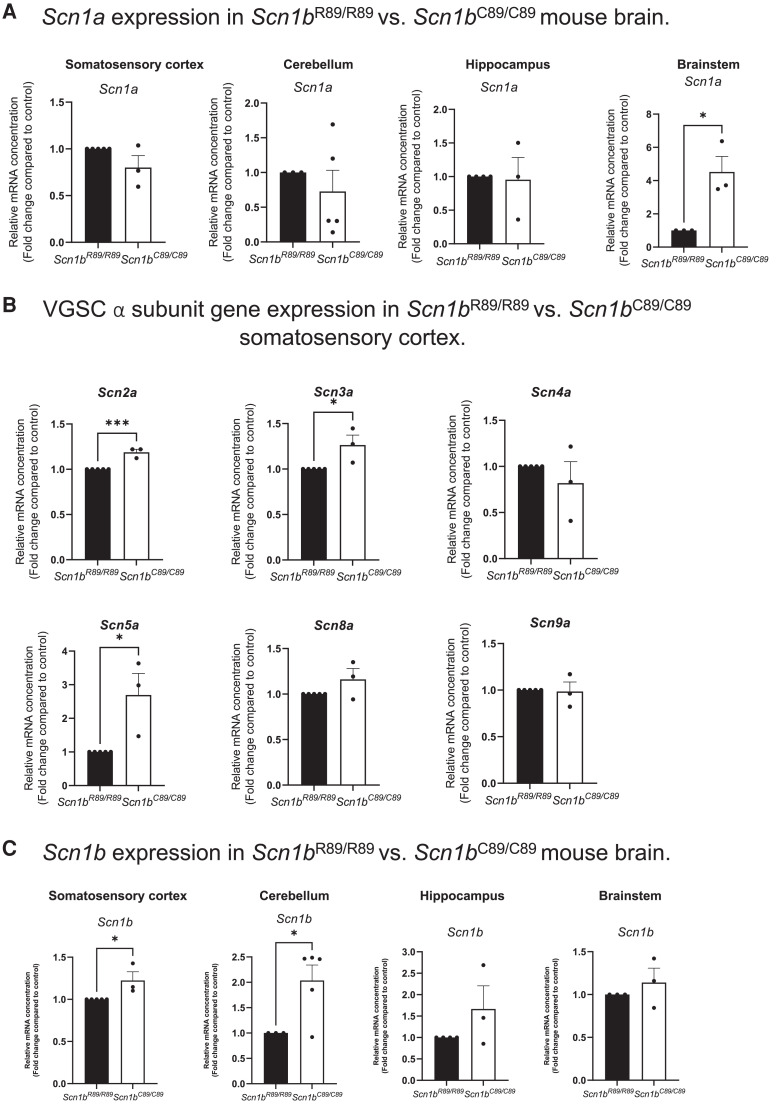
**Differential VGSC α and β subunit mRNA expression in P15-18 *Scn1b^R89/R89^* and *Scn1b^C89/C89^*mouse brains. (A)** S*cn1a* expression in *Scn1b^R89/R89^* versus *Scn1b^C89/C89^* mouse brain. Relative expression of *Scn1a* in *Scn1b^R89/R89^* versus *Scn1b^C89/C89^* mouse somatosensory cortex, cerebellum, hippocampus and brainstem. *Scn1a* gene expression was significantly increased in *Scn1b^C89/C89^* brainstem compared to *Scn1b^R89/R89^* (*P* *< 0.05*); however, there was no change in *Scn1a* mRNA between genotypes in the cortex, cerebellum or hippocampus. **(B)** VGSC α subunit gene expression in *Scn1b^R89/R89^* versus *Scn1b^C89/C89^* somatosensory cortex. Relative expression of *Scn2a*, *Scn3a*, *Scn4a*, *Scn5a*, *Scn8a* and *Scn9a* in *Scn1b^R89/R89^* versus *Scn1b^C89/C89^* somatosensory cortex. *Scn2a* (*P* *< 0.001*), *Scn3a* (*P* < 0.05) and *Scn5a* (*P* *< 0.05*) were significantly increased in *Scn1b^C89/C89^* cortex compared with *Scn1b^R89/R89^*. *Scn4a*, *Scn8a* and *Scn9a* mRNA expression was not different between genotypes (*P > 0.05*). **(C)** *Scn1b* expression in *Scn1b^R89/R89^* versus *Scn1b^C89/C89^* mouse brain. Relative expression of *Scn1b* in *Scn1b^R89/R89^* versus *Scn1b^C89/C89^* mouse somatosensory cortex, cerebellum, hippocampus and brainstem. *Scn1b* gene expression was significantly increased in *Scn1b^C89/C89^* cortex (*P* < 0.05) and cerebellum (*P* < 0.05) compared with *Scn1b^R89/R89^*, however, there was no change in the hippocampus or brainstem. Statistical significance was determined using Student’s *t*-test (*P < 0.05*). Data are represented as the mean ± SEM. *Scn1b^R89/R89^*: *n* = 3–5, *Scn1b^C89/C89^*: *n* = 3–5. Male and female mice were used in all experiments.

*Scn1b^C89/C89^* mice, in which a β1-ICD is generated (Fig. 2), showed differential VGSC α subunit mRNA expression compared with *Scn1b^−/−^* mice, in which the β1-ICD signalling cascade is absent. *Scn1b^C89/C89^* mice had significantly increased *Scn1a* mRNA abundance in the brainstem compared with *Scn1b^R89/R89^* (*P* < 0.05); however, in contrast to *Scn1b^−/−^* mice, there were no changes in *Scn1a* mRNA levels detected in the somatosensory cortex, cerebellum or hippocampus (Fig. 5A). Instead, *Scn2a* (*P* < 0.001), *Scn3a* (*P* < 0.05) and *Scn5a* (*P* < 0.05) mRNA levels were increased in the somatosensory cortex of *Scn1b^C89/C89^* mice compared with *Scn1b^R89/R89^* mice (Fig. 5B). Finally, *Scn1b^C89/C89^* mice showed increased *Scn1b* mRNA abundance in the somatosensory cortex (*P* < 0.05) and cerebellum (*P* < 0.05) compared with *Scn1b^R89/R89^*, with no changes in the hippocampus or brainstem (Fig. 5C), suggesting compensatory upregulation of the mutant gene in specific brain areas.

### *Scn1b^C89/C89^* pups have increased susceptibility to hyperthemia-induced seizures

**Figure 6:**
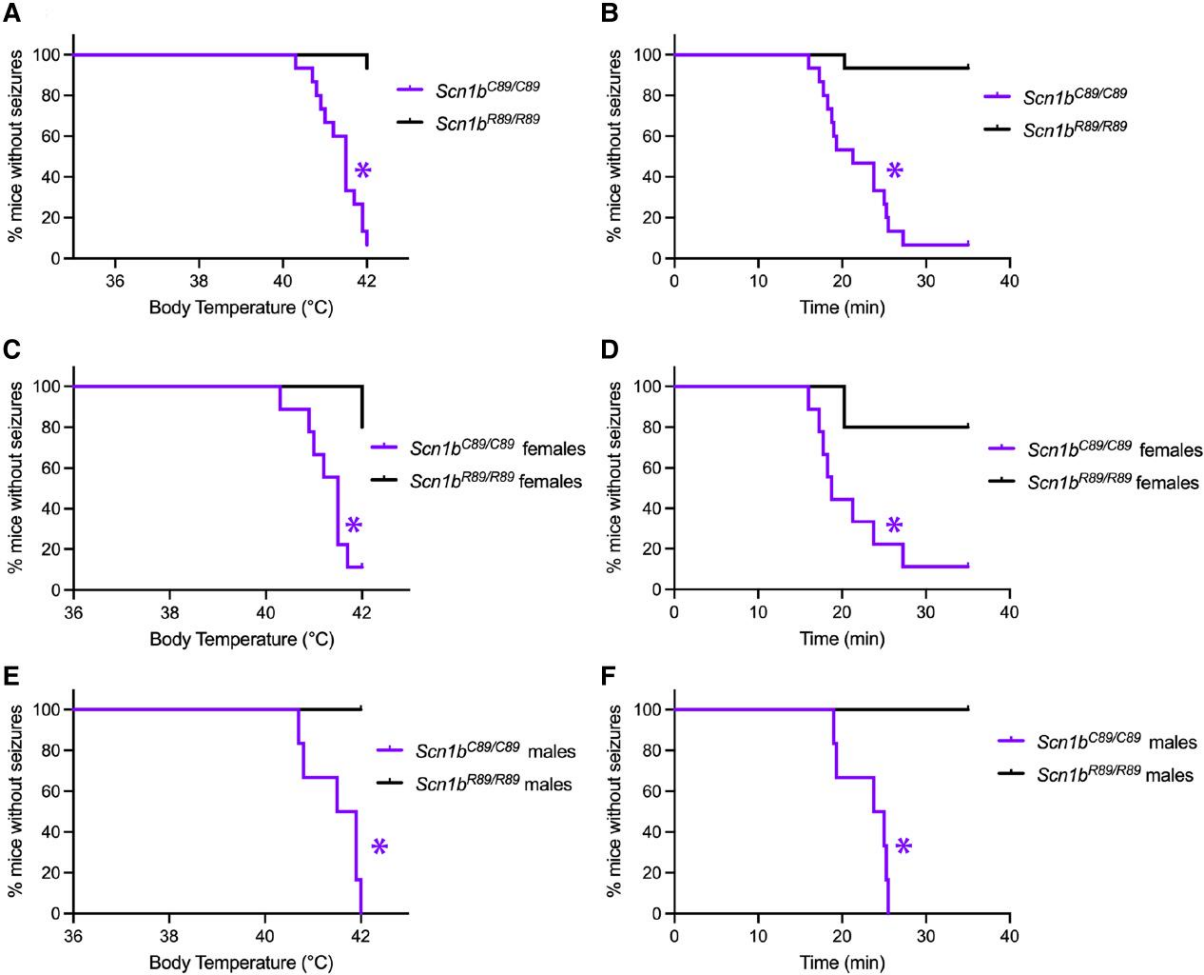
***Scn1b^C89/C89^* mice are more susceptible to hyperthermia-induced seizures than *Scn1b^R89/R89^* littermates at P15.** Behavioural seizures were observed and recorded by an investigator blinded to genotype. Seizures were induced as described in Methods. Kaplan–Meier curves showing first observed seizure for all mice (female and male) **(A),** for female mice only **(C),** or for male mice only **(E)** in relation to temperature. Survival curves to first observed seizure for all mice **(B),** for female mice only **(D),** or for male mice only **(F)** in relation to time. For all panels: *Scn1b^R89/R89^* mouse data = black; *Scn1b^C89/C89^* mouse data = purple. The numbers of mice used were: *Scn1b^R89/R89^ n* = 15 (5 female and 10 male), *Scn1b^C89/C89^ n* = 15 (9 female and 6 male). *P < 0.05 (Log-rank Mantel–Cox test).

Because the proband had frequent febrile seizures as an infant, we compared hyperthermia seizure susceptibility between *Scn1b^R89/R89^* and *Scn1b^C89/C89^* mice at P15. *Scn1b^C89/C89^* pups had convulsive seizures at significantly lower temperatures than *Scn1b^R89/R89^* littermates (Fig. 6A; in all panels *Scn1b^R89/R89^* indicated in black; *Scn1b^C89/C89^* indicated in purple). In addition, *Scn1b^C89/C89^* pups showed reduced latency to first seizure compared with *Scn1b^R89/R89^* animals (Fig. 6B). When the hyperthermia seizure data were separated by sex, no differences in temperature or latency were observed between *Scn1b^C89/C89^* male and female pups (Fig. 6C–F). These results indicate that, similar to the proband, the biallelic *Scn1b*-p.R89C mutation confers increased hyperthermia seizure sensitivity to paediatric animals.

### *Scn1b^C89/C89^* mice have spontaneous seizures

**Figure 7:**
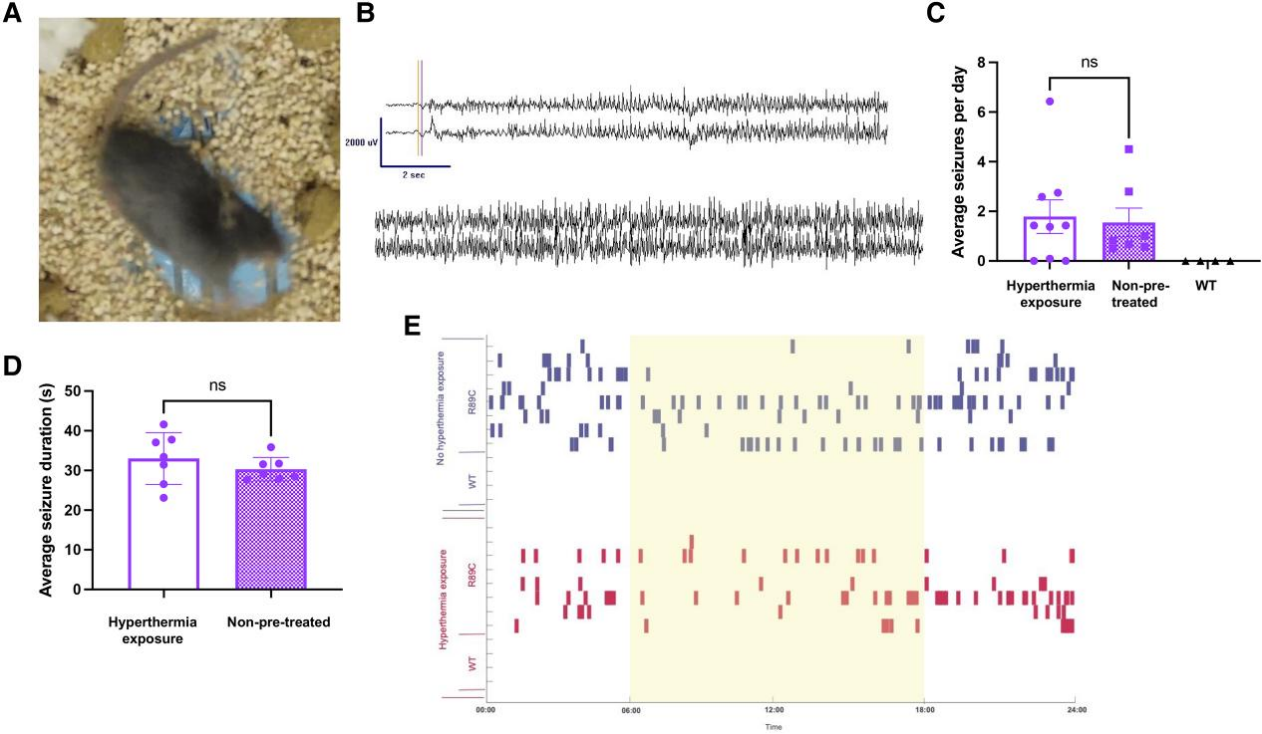
***Scn1b^C89/C89^* mice have spontaneous generalized seizures. (A)** Still photo from Video 1 showing a spontaneous generalized seizure in a *Scn1b^C89/C89^* mouse. **(B)** EEG trace showing a generalized seizure in a *Scn1b^C89/C89^* mouse displayed in a referential montage of L parietal-Ref (top trace) and R parietal-Ref (bottom trace). **(C)** Average seizures per day in young adult (P60–90) *Scn1b^C89/C89^* mice that were exposed to hyperthermia at P15 (clear bar, *n* = 8) versus mice that had no pre-exposure (purple bar, *n* = 8) versus *Scn1b^R89/R89^* mice (WT, black symbols, *n* = 4). There was no significant difference between seizure frequency between hyperthermia exposure and non-pretreated groups (*P = 0.80*, unpaired *t*-test). **(D)** Average seizure duration in *Scn1b^C89/C89^* mice that were pre-exposed to hyperthermia at P15 (clear bar, *n* = 8) versus mice that had no pre-exposure (purple bar, *n* = 8). There were no significant differences between groups (unpaired *t*-test). **(E)** Raster plot showing time of seizure occurrence for young adult *Scn1b^C89/C89^* (R89C) or *Scn1b^R89/R89^* mice (WT) mice during the light (yellow) and dark (white) cycles during the entirety of the recording period (8–14 days). Blue: Non-pretreated mice. Red: Mice that were pre-exposed to hyperthermia at P15.

Continuous video/EEG recordings showed that *Scn1b^C89/C89^* mice have spontaneous convulsive seizures observed that begin as early as P13 (video recording alone) with ictal electrographic patterns that were characterized by a sudden-onset bilateral spike, followed by attenuation and increasing fast activity, with spike discharges that increased in frequency and amplitude before sudden cessation, background attenuation and bilateral delta slowing. The majority of the electrographic seizures resulted in a Racine Grade 4–6 clinical seizure (Fig. 7A and B; Video 1). No seizures were observed in *Scn1b^R89/R89^* (Fig. 7E) or *Scn1b^R89/C89^* mice (not shown).

Because the proband had early-life febrile seizures that later became more severe and afebrile, we asked whether early-life (P15) exposure to hyperthermia would result in increased spontaneous seizure frequency or duration in *Scn1b^C89/C89^* mice later in life. We found no differences in behavioural seizure frequency or duration in *Scn1b^C89/C89^* mice that had undergone early-life hyperthermia compared with those that had not (Fig. 7 C and D). A raster plot showing time of spontaneous seizures during light (yellow) versus dark (white) cycles during the entirety of the recording period (8–14 days) for *Scn1b^R89/R89^* mice, hyperthermia pre-treated *Scn1b^C89/C89^* mice (red) and non–pre-treated *Scn1b^C89/C89^* mice (blue) is shown in Fig. 7E. As expected, *Scn1b^R89/R89^* (WT) mice did not exhibit seizures. We found no differences in seizure frequency between hyperthermia pre-treated and non–pre-treated *Scn1b^C89/C89^* mice; however, for both groups there were significantly more seizures during the dark cycle (Fig. 7E and Table 1).

**Table 1:** Seizure frequency in *Scn1b^C89/C89^* mice separated by day/night cycle (06:00–18:00 and 18:00–06:00)

Time cycle	Average	SD	*t*-test
06:00–18:00 light	0.45	0.65	0.0139*
18:00–6:00 dark	0.82	1.07	

Values show average seizure frequency per day for *Scn1b^C89/C89^* mice separated by time group. Paired sample one-tailed *t*-test was performed to compare the time groups. SD, standard deviation. *Significance defined as *P* < 0.05.

## Discussion

DEEs such as DS are devastating to families because of the high degree of neurodevelopmental compromise, including developmental delay, cognitive decline and intellectual disability.^51^ Most concerning are the severe seizures and high risk of SUDEP.^52,53^ While the majority of DS cases are linked to variants in *SCN1A* that result in haploinsufficiency,^51^ a growing list of *SCN1B* biallelic variants is also linked to DS or to the more severe early infantile DEE (both classified as DEE52).^6-8,54^ Because *SCN1B* is expressed in all excitable tissues, e.g. heart, in addition to brain, and because *SCN1B* variants are linked to cardiac arrhythmias in addition to epilepsy in human patients, we have proposed that the mechanism of SUDEP in DEE52 includes cardiac arrhythmias in addition to seizures.^14,55-57^ *Scn1b*^−/−^ mice have a phenotype that is more severe than DS, with early-onset seizures of multiple aetiologies, delayed maturation of inhibitory signalling in brain, atrial and ventricular cardiac arrhythmias and death in 100% of animals prior to weaning.^26,27,56-58^

A critical next step in our ability to make correct genetic diagnoses and to discover novel treatments for DEE52 patients, including developing strategies for SUDEP prevention, is to understand whether *Scn1b^−/−^* mice accurately model human DEE52 and to determine if all *SCN1B* pathogenic variants are LOF. Heterologous studies of DEE52 variants expressed as mutant β1 subunit polypeptides have provided important insights. For example, we showed that the mutant β1-p.R125C protein is retained intracellularly in heterologous cells, predicting LOF.^7^ In contrast, the mutant β1-p.R85C is expressed at the plasma membrane in heterologous cells but does not modify Nav1.1-generated *I_Na_*, suggesting partial, but not complete, LOF.^8^ However, while heterologous expression systems are valuable, they cannot replicate native neurons, much less model complex brain networks, with cell type-specific expression of an array of ion channel subtypes, neurotransmitters and intracellular signalling molecules. Importantly, heterologous systems also do not provide information on neuro-cardiac mechanisms of SUDEP. Thus, the development and validation of transgenic animal models of biallelic human DEE52 variants is essential to our goal of identifying novel therapeutics for *SCN1B*-linked DEE.

Here, we show that *Scn1b^C89/C89^* mice have normal body weights and a premature mortality rate of ∼20%, compared with severely reduced body weight and 100% mortality in *Scn1b^−/−^* mice. Unlike *Scn1b^−/−^* mice, β1 subunit polypeptides are expressed in *Scn1b^C89/C89^* mice and heterologous expression studies predict cell surface localization. The predicted mutant β1 polypeptide, β1-p.R89C, modulates *I_Na_* density generated by Nav1.6 in heterologous cells but has no significant effects on *I_Na_* density generated by Nav1.1 or Nav1.5, suggesting VGSC α subunit selective effects *in vivo*. We found that VGSC α subunit mRNA abundance is differentially altered in *Scn1b^C89/C89^* brains. While *Scn1a* mRNA abundance in somatosensory cortex is normal, levels of *Scn2a*, *Scn3a* and *Scn5a* mRNA are increased relative to *Scn1b^R89/R89^* littermates, which may contribute to hyperexcitability. *Scn1b* mRNA abundance is increased in *Scn1b^C89/C89^* brains compared to *Scn1b^R89/R89^* littermates, suggesting a compensatory mechanism in neurons to attempt to overcome LOF effects. As expected, *Scn1b^C89/C89^* pups are more susceptible to hyperthermia-induced seizures than *Scn1b^R89/R89^* littermates. In addition, EEG recordings detected epileptic discharges in young adult *Scn1b^C89/C89^* mice that coincided with convulsive seizures and myoclonic jerks. *Scn1b^−/−^* and *Scn1b^C89/C89^* pups begin to exhibit convulsive seizures at similar time points, ∼P13.^26^ Because the proband in our study experienced frequent early-life febrile seizures, we compared seizure onset, frequency and duration in a subset of young adult *Scn1b^C89/C89^* mice that had been exposed to hyperthermia at P15 versus a subset that were not exposed; however, this treatment did not result in increased frequency or duration of spontaneous seizures. For hyperthermia-exposed and non-exposed young adult *Scn1b^C89/C89^* mice, the spontaneous seizure pattern was diurnal, occurring with higher frequency during the dark cycle. Taken together, our results suggest that the *SCN1B-*c.265C > T variant does not result in complete *SCN1B* LOF. *Scn1b^C89/C89^* mice more accurately model partial LOF DEE52 variants than *Scn1b^−/−^* mice, which model complete LOF variants. The combined results from these two models will enhance our ability to identify novel therapeutics for DEE52 patients.

Interestingly, we found *Scn1b^−/−^*, but not *Scn1b^C89/C89^,* mouse somatosensory cortex to be haploinsufficient for *Scn1a*, with reduced Nav1.1 protein in whole brain. We propose that the absence of β1-ICD formation through the RIP cascade^22^ results in dysregulation of *Scn1a* expression in *Scn1b^−/−^* cortical neurons, with subsequent disruption of excitatory:inhibitory balance. These data are consistent with our previous report of reduced *I_Na_* density and hypoexcitability of parvalbumin-positive fast-spiking interneurons in *Scn1b^−/−^* cortex.^27^ This observation may provide at least a partial explanation for the increased severity of the *Scn1b* null model via disrupted transcriptional regulation of another VGSC gene implicated in DS, resulting in an effective double-hit mutation.

The work presented here is the first report of a transgenic mouse model of DEE52. Previous work from the Petrou group characterized the variant *SCN1B*-p.C121W in transgenic mice^59^; however, this variant is associated with genetic epilepsy with febrile seizures plus (GEFS+) in monoallelic patients and has not yet been reported in a biallelic patient with DEE52. We chose the *SCN1B-*c.265C>T variant for the present work because of its identification in three patients in two unrelated DEE52 families. Development of this animal model will allow future studies of epilepsy mechanisms, cardiac arrhythmia, cardiac myocyte excitability and neuro-cardiac contributions to SUDEP. The variability in phenotypic severity between the identified probands suggests genetic background effects, which can be studied in the future by crossing this novel mouse line to various background strains. Importantly, this new work suggests that not all *SCN1B* DEE variants result in complete LOF. While *Scn1b^−/−^* mice remain a valuable model for complete LOF variants, this new mouse model is an important new tool in understanding how *SCN1B* partial LOF results in DEE52.

## Supplementary Material

fcad283_Supplementary_Data

## Data Availability

The data that support the findings of this study are available from the corresponding author, upon reasonable request.

## References

[1] Brunklaus A, Feng T, Brunger T, et al Gene variant effects across sodium channelopathies predict function and guide precision therapy. Brain. 2022;145(12):4275–4286. 35037686 10.1093/brain/awac006 PMC9897196

[2] Dravet C, Bureau M, Oguni H, Fukuyama Y, Cokar O. Severe myoclonic epilepsy in infancy (Dravet syndrome). Adv Neurol. 2005;95:71–102. 15508915

[3] Guerrini R, Aicardi J. Epileptic encephalopathies with myoclonic seizures in infants and children (severe myoclonic epilepsy and myoclonic-astatic epilepsy). J Clin Neurophysiol. 2003;20(6):449–461. 14734934 10.1097/00004691-200311000-00007

[4] Claes L, Del-Favero J, Ceulemans B, Lagae L, Van Broeckhoven C, De Jonghe P. De novo mutations in the sodium-channel gene SCN1A cause severe myoclonic epilepsy of infancy. Am J Hum Genet. 2001;68(6):1327–1332. 11359211 10.1086/320609 PMC1226119

[5] Meisler MH, Kearney JA. Sodium channel mutations in epilepsy and other neurological disorders. J Clin Invest. 2005;115(8):2010–2017. 16075041 10.1172/JCI25466 PMC1180547

[6] Ogiwara I, Nakayama T, Yamagata T, et al A homozygous mutation of voltage-gated sodium channel beta(I) gene SCN1B in a patient with Dravet syndrome. Epilepsia. 2012;53(12):e200-3. 23148524 10.1111/epi.12040

[7] Patino GA, Claes LR, Lopez-Santiago LF, et al A functional null mutation of SCN1B in a patient with Dravet syndrome. J Neurosci. 2009;29(34):10764–10778. 19710327 10.1523/JNEUROSCI.2475-09.2009 PMC2749953

[8] Aeby A, Sculier C, Bouza AA, et al SCN1B-linked early infantile developmental and epileptic encephalopathy. Ann Clin Transl Neurol. 2019:6;2354–2367. 31709768 10.1002/acn3.50921 PMC6917350

[9] Ramadan W, Patel N, Anazi S, et al Confirming the recessive inheritance of SCN1B mutations in developmental epileptic encephalopathy. Clin Genet. 2017:92;327–331. 28218389 10.1111/cge.12999

[10] Catterall WA. Voltage-gated sodium channels at 60: Structure, function and pathophysiology. J Physiol. 2012;590(Pt 11):2577–2589. 22473783 10.1113/jphysiol.2011.224204 PMC3424717

[11] Hartshorne RP, Catterall WA. Purification of the saxitoxin receptor of the sodium channel from rat brain. Proc Natl Acad Sci USA. 1981;78:4620–4624. 6270687 10.1073/pnas.78.7.4620 PMC319845

[12] Messner DJ, Catterall WA. The sodium channel from rat brain. Separation and characterization of subunits. JBiolChem. 1985;260:10597–10604. 2411726

[13] Brackenbury WJ, Isom LL. Na channel beta subunits: Overachievers of the ion channel family. Front Pharmacol. 2011;2:53. 22007171 10.3389/fphar.2011.00053 PMC3181431

[14] Lin X, O'Malley H, Chen C, et al Scn1b deletion leads to increased tetrodotoxin-sensitive sodium current, altered intracellular calcium homeostasis and arrhythmias in murine hearts. J Physiol. 2015;593(6):1389–1407. 25772295 10.1113/jphysiol.2014.277699 PMC4376420

[15] O’Malley HA, Isom LL. Sodium channel beta subunits: Emerging targets in channelopathies. Annu Rev Physiol. 2015;77:481–504. 25668026 10.1146/annurev-physiol-021014-071846 PMC4817109

[16] Isom LL, Catterall WA. Na^+^ channel subunits and Ig domains. Nature. 1996;383:307–308. 10.1038/383307b0 8848042

[17] Kruger LC, O'Malley HA, Hull JM, Kleeman A, Patino GA, Isom LL. beta1-C121W is down but not out: Epilepsy-associated Scn1b-C121W results in a deleterious gain-of-function. J Neurosci. 2016;36(23):6213–6224. 27277800 10.1523/JNEUROSCI.0405-16.2016 PMC4899524

[18] Marionneau C, Carrasquillo Y, Norris AJ, et al The sodium channel accessory subunit Navbeta1 regulates neuronal excitability through modulation of repolarizing voltage-gated K(+) channels. J Neurosci. 2012;32(17):5716–5727. 22539834 10.1523/JNEUROSCI.6450-11.2012 PMC3347704

[19] Deschenes I, Armoundas AA, Jones SP, Tomaselli GF. Post-transcriptional gene silencing of KChIP2 and Navbeta1 in neonatal rat cardiac myocytes reveals a functional association between Na and Ito currents. J Mol Cell Cardiol. 2008;45(3):336–346. 18565539 10.1016/j.yjmcc.2008.05.001 PMC2580777

[20] Deschenes I, DiSilvestre D, Juang GJ, Wu RC, An WF, Tomaselli GF. Regulation of Kv4.3 current by KChIP2 splice variants: A component of native cardiac I(to)? Circulation. 2002;106(4):423–429. 12135940 10.1161/01.cir.0000025417.65658.b6

[21] Nguyen HM, Miyazaki H, Hoshi N, et al Modulation of voltage-gated K+ channels by the sodium channel beta1 subunit. Proc Natl Acad Sci USA. 2012;109(45):18577–18582. 23090990 10.1073/pnas.1209142109 PMC3494885

[22] Bouza AA, Edokobi N, Hodges SL, et al Sodium channel beta1 subunits participate in regulated intramembrane proteolysis-excitation coupling. JCI Insight. 2021;6:e141776. 33411695 10.1172/jci.insight.141776 PMC7934843

[23] Wong HK, Sakurai T, Oyama F, et al beta subunits of voltage-gated sodium channels are novel substrates of beta-site amyloid precursor protein-cleaving enzyme (BACE1) and gamma-secretase. J Biol Chem. 2005;280(24):23009–23017. 15824102 10.1074/jbc.M414648200

[24] Kim DY, Carey BW, Wang H, et al BACE1 regulates voltage-gated sodium channels and neuronal activity. Nat Cell Biol. 2007;9(7):755–764. 17576410 10.1038/ncb1602 PMC2747787

[25] Darras N, Ha TK, Rego S, et al Developmental and epileptic encephalopathy in two siblings with a novel, homozygous missense variant in SCN1B. Am J Med Genet A. 2019;179(11):2190–2195. 31465153 10.1002/ajmg.a.61344

[26] Chen C, Westenbroek RE, Xu X, et al Mice lacking sodium channel beta1 subunits display defects in neuronal excitability, sodium channel expression, and nodal architecture. J Neurosci. 2004;24(16):4030–4042. 15102918 10.1523/JNEUROSCI.4139-03.2004 PMC6729427

[27] Hull JM, O'Malley HA, Chen C, et al Excitatory and inhibitory neuron defects in a mouse model of Scn1b-linked EIEE52. Ann Clin Transl Neurol. 2020;7(11):2137–2149. 32979291 10.1002/acn3.51205 PMC7664274

[28] Haeussler M, Schonig K, Eckert H, et al Evaluation of off-target and on-target scoring algorithms and integration into the guide RNA selection tool CRISPOR. Genome Biol. 2016;17(1):148. 27380939 10.1186/s13059-016-1012-2 PMC4934014

[29] Basila M, Kelley ML, Smith AVB. Minimal 2'-O-methyl phosphorothioate linkage modification pattern of synthetic guide RNAs for increased stability and efficient CRISPR-Cas9 gene editing avoiding cellular toxicity. PLoS One. 2017;12(11):e0188593. 29176845 10.1371/journal.pone.0188593 PMC5703482

[30] Hendel A, Bak RO, Clark JT, et al Chemically modified guide RNAs enhance CRISPR-Cas genome editing in human primary cells. Nat Biotechnol. 2015;33(9):985–989. 26121415 10.1038/nbt.3290 PMC4729442

[31] Slaymaker IM, Gao L, Zetsche B, Scott DA, Yan WX, Zhang F. Rationally engineered Cas9 nucleases with improved specificity. Science. 2016;351(6268):84–88. 26628643 10.1126/science.aad5227 PMC4714946

[32] Sakurai T, Watanabe S, Kamiyoshi A, Sato M, Shindo T. A single blastocyst assay optimized for detecting CRISPR/Cas9 system-induced indel mutations in mice. BMC Biotechnol. 2014;14:69. 25042988 10.1186/1472-6750-14-69 PMC4118159

[33] Brinkman EK, Chen T, Amendola M, van Steensel B. Easy quantitative assessment of genome editing by sequence trace decomposition. Nucleic Acids Res. 2014;42(22):e168. 25300484 10.1093/nar/gku936 PMC4267669

[34] Doench JG, Fusi N, Sullender M, et al Optimized sgRNA design to maximize activity and minimize off-target effects of CRISPR-Cas9. Nat Biotechnol. 2016;34(2):184–191. 26780180 10.1038/nbt.3437 PMC4744125

[35] Anderson KR, Haeussler M, Watanabe C, et al CRISPR off-target analysis in genetically engineered rats and mice. Nat Methods. 2018;15(7):512–514. 29786090 10.1038/s41592-018-0011-5 PMC6558654

[36] Marusyk R, Sergeant A. A simple method for dialysis of small-volume samples. Anal Biochem. 1980;105(2):403–404. 7457844 10.1016/0003-2697(80)90477-7

[37] Paquet D, Kwart D, Chen A, et al Efficient introduction of specific homozygous and heterozygous mutations using CRISPR/Cas9. Nature. 2016; 533(7601):125–129. 27120160 10.1038/nature17664

[38] Van Keuren ML, Gavrilina GB, Filipiak WE, Zeidler MG, Saunders TL. Generating transgenic mice from bacterial artificial chromosomes: Transgenesis efficiency, integration and expression outcomes. Transgenic Res. 2009;18(5):769–785. 19396621 10.1007/s11248-009-9271-2 PMC3016422

[39] Racine RJ. Modification of seizure activity by electrical stimulation: II. Motor seizure. Electroenceph Clin Neurophysiol. 1972;32:281–294. 4110397 10.1016/0013-4694(72)90177-0

[40] Kane N, Acharya J, Benickzy S, *et al*. A revised glossary of terms most commonly used by clinical electroencephalographers and updated proposal for the report format of the EEG findings. Revision 2017. *Clin Neurophysiol Pract.* 2017;2:170-185. 10.1016/j.cnp.2017.07.002 PMC6123891 30214992

[41] Isom LL, Scheuer T, Brownstein AB, Ragsdale DS, Murphy BJ, Catterall WA. Functional co-expression of the b1 and type IIA a subunits of sodium channels in a mammalian cell line. J Biol Chem. 1995;270:3306–3312. 7852416 10.1074/jbc.270.7.3306

[42] Sutkowski EM, Catterall WA. Beta 1 subunits of sodium channels. Studies with subunit-specific antibodies. J Biol Chem. 1990;265(21):12393–12399. 2165060

[43] Johnson D, Montpetit ML, Stocker PJ, Bennett ES. The sialic acid component of the beta1 subunit modulates voltage-gated sodium channel function. J Biol Chem. 2004;279(43):44303–44310. 15316006 10.1074/jbc.M408900200

[44] Calhoun JD, Isom LL. The role of non-pore-forming beta subunits in physiology and pathophysiology of voltage-gated sodium channels. Handb Exp Pharmacol. 2014;221:51–89. 24737232 10.1007/978-3-642-41588-3_4

[45] Watanabe H, Darbar D, Kaiser DW, et al Mutations in sodium channel β1- and β2-subunits associated with atrial fibrillation. Circ Arrhythm Electrophysiol. 2009;2(3):268–275. 19808477 10.1161/CIRCEP.108.779181 PMC2727725

[46] Watanabe H, Koopmann TT, Le Scouarnec S, et al Sodium channel beta1 subunit mutations associated with Brugada syndrome and cardiac conduction disease in humans. J Clin Invest. 2008;118(6):2260–2268. 18464934 10.1172/JCI33891 PMC2373423

[47] Yuan L, Koivumaki JT, Liang B, et al Investigations of the Navbeta1b sodium channel subunit in human ventricle; functional characterization of the H162P Brugada syndrome mutant. Am J Physiol Heart Circ Physiol. 2014;306(8):H1204–H1212. 24561865 10.1152/ajpheart.00405.2013

[48] Martinez-Moreno R, Selga E, Riuro H, et al An SCN1B variant affects both cardiac-type (NaV1.5) and brain-type (NaV1.1) sodium currents and contributes to complex concomitant brain and cardiac disorders. Front Cell Dev Biol. 2020;8:528742. 33134290 10.3389/fcell.2020.528742 PMC7550680

[49] Scala M, Efthymiou S, Sultan T, et al Homozygous SCN1B variants causing early infantile epileptic encephalopathy 52 affect voltage-gated sodium channel function. Epilepsia. 2021;62(6):e82–e87. 33901312 10.1111/epi.16913 PMC8585727

[50] Mistry AM, Thompson CH, Miller AR, Vanoye CG, George AL Jr, Kearney JA. Strain- and age-dependent hippocampal neuron sodium currents correlate with epilepsy severity in Dravet syndrome mice. Neurobiol Dis. 2014;65:1–11. 24434335 10.1016/j.nbd.2014.01.006 PMC3968814

[51] Dravet C. The core Dravet syndrome phenotype. Epilepsia. 2011;52(Suppl 2):3–9. 10.1111/j.1528-1167.2011.02994.x 21463272

[52] Donner E, Buchhalter J. Commentary: It's time to talk about SUDEP. Epilepsia. 2014;55(10):1501–1503. 25323494 10.1111/epi.12794

[53] Hirsch LJ, Donner EJ, So EL, et al Abbreviated report of the NIH/NINDS workshop on sudden unexpected death in epilepsy. Neurology. 2011;76(22):1932–1938. 21543734 10.1212/WNL.0b013e31821de7de PMC3115809

[54] Zhu Z, Bolt E, Newmaster K, et al SCN1B Genetic variants: A review of the spectrum of clinical phenotypes and a report of early myoclonic encephalopathy. Children (Basel). 2022;9(10);1507. 36291443 10.3390/children9101507 PMC9600564

[55] Meadows LS, Chen C, Speelman AI, Malhotra JD, Isom LL. Characterization of cardiac sodium channel function in b1 subunit null mice. *Program No 2629 2004 Abstract Viewer/Itinerary Planner*. Society for Neuroscience; 2004.

[56] Lopez-Santiago LF, Meadows LS, Ernst SJ, et al Sodium channel Scn1b null mice exhibit prolonged QT and RR intervals. J Mol Cell Cardiol. 2007;43(5):636–647. 17884088 10.1016/j.yjmcc.2007.07.062 PMC2099572

[57] Ramos-Mondragon R, Edokobi N, Hodges SL, et al Neonatal Scn1b-null mice have sinoatrial node dysfunction, altered atrial structure, and atrial fibrillation. JCI Insight. 2022;7(10):e152050. 35603785 10.1172/jci.insight.152050 PMC9220823

[58] Yuan Y, O'Malley HA, Smaldino MA, Bouza AA, Hull JM, Isom LL. Delayed maturation of GABAergic signaling in the Scn1a and Scn1b mouse models of Dravet Syndrome. Sci Rep. 2019;9(1):6210. 30996233 10.1038/s41598-019-42191-0 PMC6470170

[59] Wimmer VC, Harty RC, Richards KL, et al Sodium channel beta1 subunit localizes to axon initial segments of excitatory and inhibitory neurons and shows regional heterogeneity in mouse brain. J Comp Neurol. 2015;523(5):814–830. 25421039 10.1002/cne.23715

