# Correction to: Epilepsy and sudden unexpected death in epilepsy in a mouse model of human *SCN1B*-linked developmental and epileptic encephalopathy

**DOI:** 10.1093/braincomms/fcae009

**Published:** 2024-01-24

This is a correction to: Chunling Chen, Julie Ziobro, Larissa Robinson-Cooper, Samantha L Hodges, Yan Chen, Nnamdi Edokobi, Luis Lopez-Santiago, Karl Habig, Chloe Moore, Joe Minton, Sabrina Bramson, Caroline Scheuing, Noor Daddo, Katalin Štěrbová, Sarah Weckhuysen, Jack M Parent, Lori L Isom, Epilepsy and sudden unexpected death in epilepsy in a mouse model of human *SCN1B*-linked developmental and epileptic encephalopathy, *Brain Communications*, Volume 5, Issue 6, 2023, fcad283, https://doi.org/10.1093/braincomms/fcad283

In the originally published version of this manuscript, figure 3 was incorrect.

The manuscript has now been updated with the correct figure.

