# Supplementary material for: Epilepsy and sudden unexpected death in epilepsy in a mouse model of human SCN1B-linked developmental and epileptic encephalopathy
Source: Brain Commun. 2023 Oct 20;5(6):fcad283. doi: 10.1093/braincomms/fcad283 (PMC10903178; doi:10.1093/braincomms/fcad283)
Supplement: fcad283_Supplementary_Data [file fcad283_Supplementary_Data.pdf]

SUPPLEMENTARY MATERIAL

Epilepsy and SUDEP in a mouse model of human *SCN1B*-linked Developmental and Epileptic Encephalopathy

Chunling Chen[1], Julie Ziobro[2], Larissa Robinson-Cooper[1], Samantha L. Hodges[1], Yan Chen[1], Nnamdi Edokobi[1], Luis Lopez-Santiago[1], Karl Habig[1], Chloe Moore[1], Joe Minton[1], Sabrina Bramson[1], Caroline Scheuing[1], Noor Daddo[5], Katalin Štěrbová[3], Sarah Weckhuysen[4,7,8,9], Jack M. Parent[5,6,10], and Lori L. Isom[1,5]

Author affiliations:
[1]Department of Pharmacology, University of Michigan Medical School, Ann Arbor, MI 48109, USA
[2]Department of Pediatrics, University of Michigan Medical School, Ann Arbor, MI 48109, USA
[3]Department of Pediatric Neurology, Charles University and Motol Hospital, Prague, Czech Republic
[4]Applied&Translational Neurogenomics Group, VIB Center for Molecular Neurology, VIB, Antwerp, Belgium
[5]Department of Neurology, University of Michigan Medical School, Ann Arbor, MI 48109, USA
[6]Michigan Neuroscience Institute, University of Michigan Medical School, Ann Arbor, MI 48109, USA
[7]Translational Neurosciences, Faculty of Medicine and Health Science, University of Antwerp, Antwerp, Belgium
[8]Department of Neurology, Antwerp University Hospital, Antwerp, Belgium
[9]µNEURO Research Centre of Excellence, University of Antwerp, Antwerp, Belgium
[10]VA Ann Arbor Healthcare System, Ann Arbor, MI 49105, USA

Supplementary Figures:

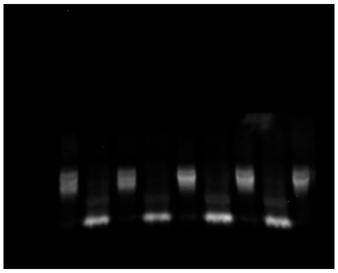

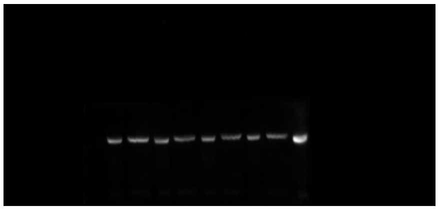

Supplementary Figure 1. Original, uncropped blots for Figure 1F.

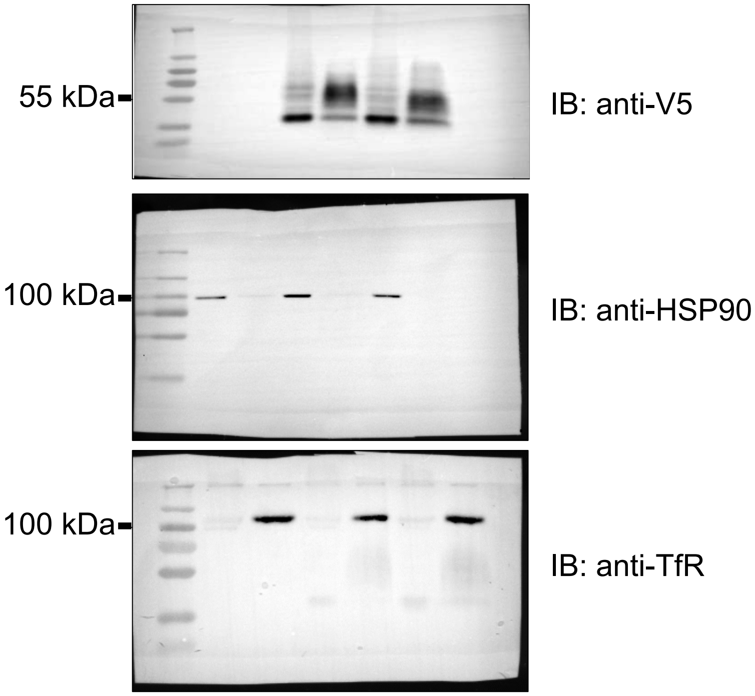

Supplementary Figure 2. Original, uncropped blots for Figure 2A.

IB: anti-V5 (β1)

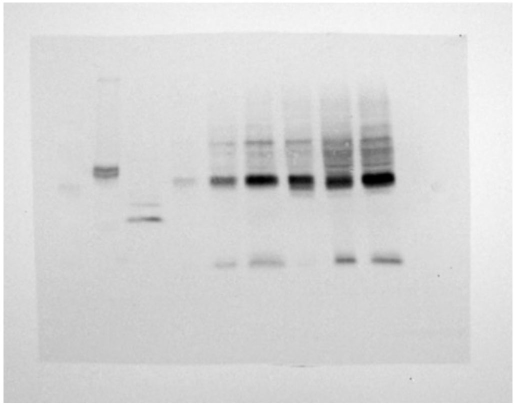

IB: anti-α-tubulin

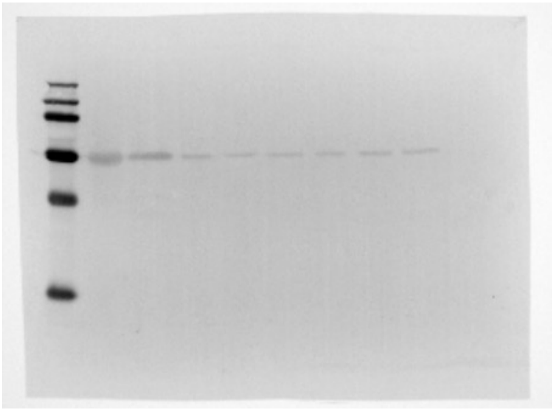

Supplementary Figure 3: Original, uncropped blots for Figure 2B.

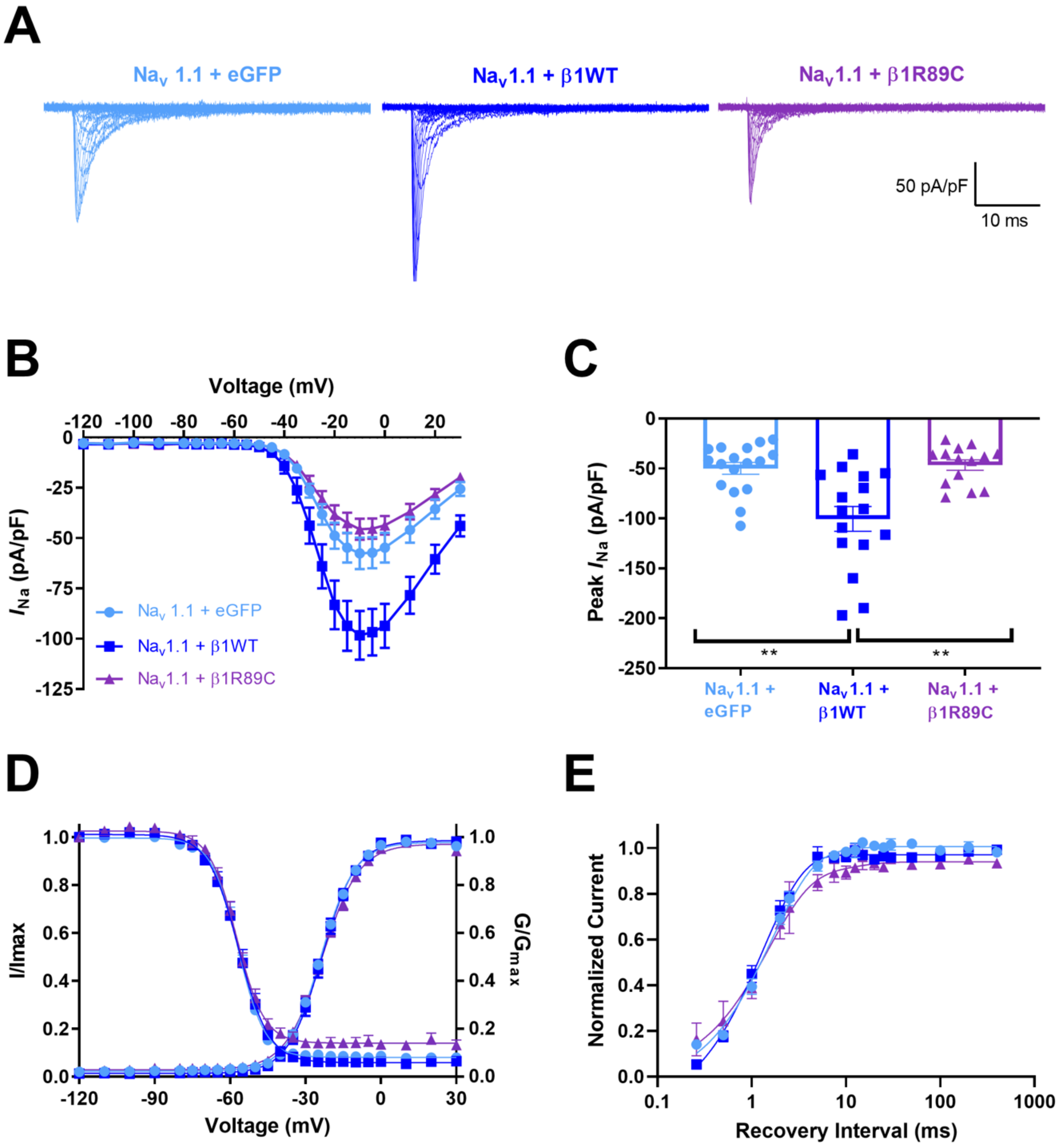

Supplementary Figure 4. β1-p.R89C does not modulate Nav1.1-generated I_{Na} density. HEK cells stably expressing human $Na_v1.1$ were transiently co-transfected with β1-WT-V5-2AeGFP (dark blue) β1-p.R89C-V5-2AeGFP (purple), or eGFP (light blue). Cells transfected with eGFP were used as negative controls. (A) Representative I_{Na} density traces. (B) $Na_v1.1$ I_{Na} current-voltage relationship. (C) I_{Na} density was increased with co-expression of β1-WT but unchanged in the

presence of β1-p.R89C. (D) No differences were observed in the mean voltage-dependent activation and inactivation curves. (E) Recovery from inactivation was expressed as the fraction of current produced by a second pulse over time following an identical pre-pulse. The data were fit to a double exponential function. Data in (B), (C), (D), and (E) are presented as means ± SEM. **$p < 0.01$ by a one-way ANOVA with Tukey's post-hoc comparison test. Dots represent individual cells.

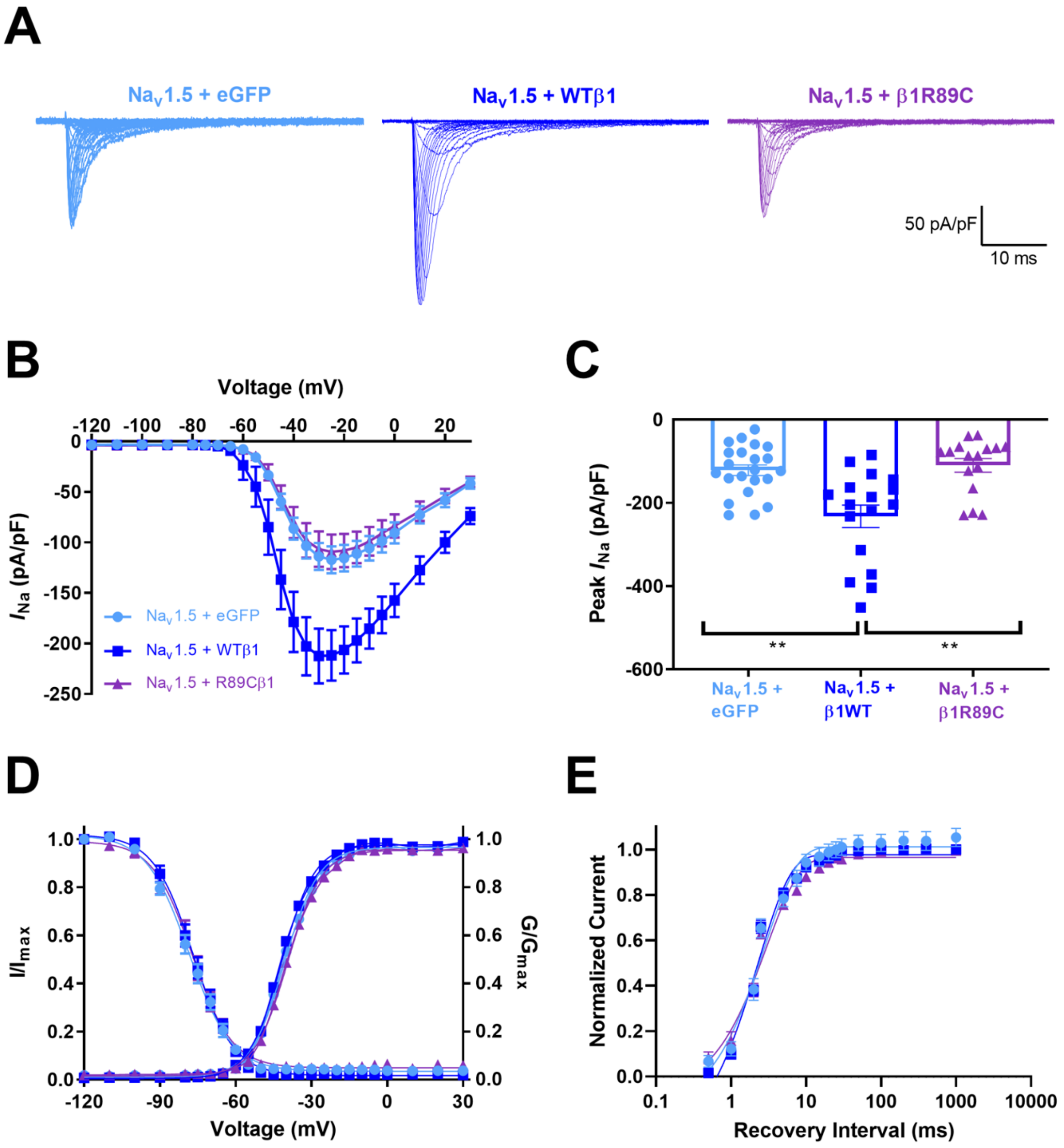

Supplementary Figure 5. β1-p.R89C does not modulate Na$_V$1.5-generated I_{Na} density. HEK cells stably expressing human Na$_V$1.5 were transiently co-transfected with β1-WT-V5-2AeGFP (dark blue) β1-p.R89C-V5-2AeGFP (purple), or eGFP (light blue). Cells transfected with eGFP were used as negative controls. (A) Representative I_{Na} density traces. I_{Na} was recorded in response to a series of voltage steps between -120 and +30mV in 5 mV increments, from a holding potential of

-120 mV for 200 msec. (B) $Na_v1.5$ I_{Na} current-voltage relationship. (C) I_{Na} density was increased with co-expression of β1-WT but unchanged in the presence of β1-p.R89C. (D) No differences were found in the mean voltage-dependent activation and inactivation curves. Data were obtained by fitting individual activation or inactivation curves to a Boltzmann equation. (E) A standard two pulse protocol was used to investigate the recovery from inactivation. Recovery from inactivation was expressed as the fraction of current produced by a second pulse over time following an identical pre-pulse. The data were fit to a double exponential function. Data in (B), (C), (D), and (E) are presented as means ± SEM. $**p < 0.01$ by a one-way ANOVA with Tukey's post-hoc comparison test. Dots represent individual cells.

Supplementary Tables

**$Na_v1.5$**	**+ eGFP**	**+ β1 WT**	**+ β1-p.R89C**
**Voltage dependence of activation**			
$V_{1/2}$ (mV)	-40.61 ± 0.24	-41.78 ± 0.39	-39.50 ± 0.24
k (mV)	6.77 ± 0.21	6.53 ± 0.34	6.65 ± 0.21
n	18	16	14
**Voltage dependence of inactivation**			
$V_{1/2}$ (mV)	-78.45 ± 0.48	-76.71 ± 0.43	-77.20 ± 0.56
h (mV)	-8.45 ± 0.40	-7.90 ± 0.37	-7.83 ± 0.48
n	18	16	14
**Recovery from Inactivation**			
τ_{fast} (ms)	7.55 ± 0.78	4.845 ± 0.60*	4.932 ± 0.51*
n	9	6	7

Supplementary Table 1. Biophysical properties of I_{Na} expressed by $Na_v1.5$. Data are presented as means ± sem. *$p < 0.05$ versus + eGFP using a one-way Anova with Tukey's post-hoc comparison test.

$Na_v1.1$	+ eGFP	+ β1 WT	+ β1-p.R89C
**Voltage dependence of activation**			
$V_{1/2}$ (mV)	-23.90 ± 0.18	-23.51 ± 0.28	-23.34 ± 0.26
k (mV)	6.81 ± 0.15	6.78 ± 0.24	7.40 ± 0.22
n	17	16	14
**Voltage dependence of inactivation**			
$V_{1/2}$ (mV)	-56.86 ± 0.25	-56.61 ± 0.40	-57.40 ± 0.40
h (mV)	-5.32 ± 0.22	-6.07 ± 0.35	-5.52 ± 0.35
n	17	16	14
**Recovery from Inactivation**			
τ_{fast} (ms)	1.17 ± 0.14	1.01 ± 0.25	1.05 ± 0.18
n	8	6	6

**Supplementary Table 2. Biophysical properties of I_{Na} expressed by Na_v1.**1. Data are presented as means ± sem. *$p < 0.05$ versus + eGFP using a one-way Anova with Tukey's post-hoc comparison test.

$Na_v1.6$	+ eGFP	+ β1 WT	+ β1-p.R89C
**Voltage dependence of activation**			
$V_{1/2}$ (mV)	-20.06 ± 0.29	-19.49 ± 0.32	-20.12 ± 0.21
k (mV)	6.36 ± 0.26	6.00 ± 0.26	5.91 ± 0.26
n	15	11	17
**Voltage dependence of inactivation**			
$V_{1/2}$ (mV)	-58.29 ± 0.64	-55.55 ± 0.78	-54.93 ± 0.41
h (mV)	-6.46 ± 0.56	-5.41 ± 0.66	-5.95 ± 0.35
n	15	11	17
**Recovery from Inactivation**			
τ_{fast} (ms)	1.42 ± 0.56	0.93 ± 0.24	2.28 ± 0.49
n	8	8	10

Supplementary Table 3. Biophysical properties of I_{Na} expressed by $Na_v1.6$. Data are presented as means ± sem. *$p < 0.05$ versus + eGFP using a one-way Anova with Tukey's post-hoc comparison test.
